# Supplementary figures and images for: Transcriptome analysis of Actinidia chinensis in response to Botryosphaeria dothidea infection
Source: PLoS One. 2020 Jan 8;15(1):e0227303. doi: 10.1371/journal.pone.0227303 (PMC6948751; doi:10.1371/journal.pone.0227303)

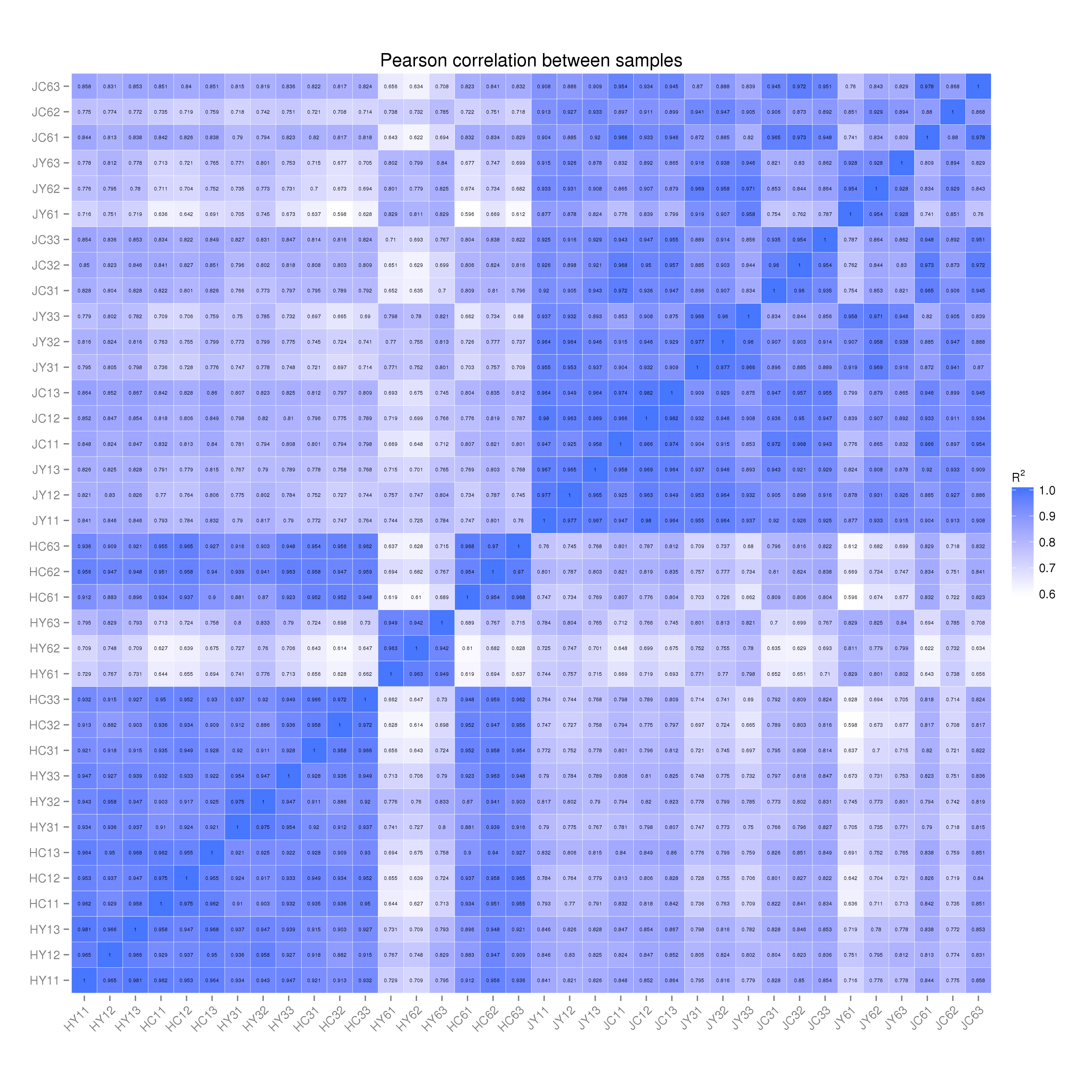

Supplement: S3 Fig — (PNG) [file pone.0227303.s003.png]
