# Supplementary material for: Transcriptome analysis of Actinidia chinensis in response to Botryosphaeria dothidea infection
Source: PLoS One. 2020 Jan 8;15(1):e0227303. doi: 10.1371/journal.pone.0227303 (PMC6948751; doi:10.1371/journal.pone.0227303)
Supplement: S1 Table — (DOCX) [file pone.0227303.s005.docx]

**S1 Table. Primers used for RT-qPCR analysis of differentially expressed genes**

| **Gene ID** | **Annotation** | **Forward primer (5’-3’)** | **Reverse primer (5’-3’)** |
| --- | --- | --- | --- |
| *Achn012851* | calcium-transporting ATPase 1 | CCAATACAACACCCCTCACC | GTGGACCGGGATCATCTTTA |
| *Achn040411* | axi 1 like protein | TTTCTCCCACTCCACTCTCG | TCGGAACCCTTCAAACCTC |
| *Achn104901* | protein phosphatase-2C | CTGGAATAGCCAAGCGACTG | ATCGTGGAAATGACGCCTAA |
| *Achn251121* | protein phosphatase-2C | CGGTTATCTCAATGGCGTTC | CTCGGTGAGGACTACCTGCT |
| *Achn327381* | calmodulin-like protein 1 | GTCGAACAACATCACCACCA | TTATCTCCGGCCATTCACC |
| *Achn372801* | GDSL esterase/lipase | AACACGGTTTGTGGTTCTCC | CCTTTATGGATTTGCCTTGC |
| *Achn386421* | protein COBRA | CACCTCAGACGACGGATTTC | GGGATTATGGTTTGCGATTG |
| *Actin* | Actin isoform B | GTGCTCAGTGGTGGTTCAA | GACGCTGTATTTCCTCTCAG |
