# Supplementary material for: Transcriptome analysis of Actinidia chinensis in response to Botryosphaeria dothidea infection
Source: PLoS One. 2020 Jan 8;15(1):e0227303. doi: 10.1371/journal.pone.0227303 (PMC6948751; doi:10.1371/journal.pone.0227303)
Supplement: S2 Table — (PDF) [file pone.0227303.s006.pdf]

S2 Table. Number of genes at different FPKM interval after significance level correction

FPKM Interval	0~1	1~3	3~15	15~60	>60
JY11	21859(48.95%)	4744(10.62%)	8910(19.95%)	6447(14.44%)	2696(6.04%)
JY12	22453(50.28%)	4516(10.11%)	8758(19.61%)	6180(13.84%)	2749(6.16%)
JY13	22618(50.65%)	4584(10.27%)	8832(19.78%)	6059(13.57%)	2563(5.74%)
JC11	21741(48.69%)	4695(10.51%)	9347(20.93%)	6434(14.41%)	2439(5.46%)
JC12	21750(48.71%)	4709(10.55%)	9266(20.75%)	6438(14.42%)	2493(5.58%)
JC13	21315(47.73%)	4642(10.40%)	9388(21.02%)	6748(15.11%)	2563(5.74%)
JY31	22738(50.92%)	4781(10.71%)	8628(19.32%)	5896(13.20%)	2613(5.85%)
JY32	22103(49.50%)	4771(10.68%)	8945(20.03%)	6107(13.68%)	2730(6.11%)
JY33	21964(49.18%)	4839(10.84%)	8928(19.99%)	6163(13.80%)	2762(6.19%)
JC31	22262(49.85%)	4521(10.12%)	9038(20.24%)	6273(14.05%)	2562(5.74%)
JC32	21323(47.75%)	4898(10.97%)	9638(21.58%)	6354(14.23%)	2443(5.47%)
JC33	21756(48.72%)	4992(11.18%)	9542(21.37%)	5865(13.13%)	2501(5.60%)
JY61	22865(51.20%)	4851(10.86%)	8594(19.24%)	5656(12.67%)	2690(6.02%)
JY62	22460(50.30%)	4739(10.61%)	8788(19.68%)	6007(13.45%)	2662(5.96%)
JY63	21438(48.01%)	4757(10.65%)	9099(20.38%)	6361(14.24%)	3001(6.72%)
JC61	21573(48.31%)	4804(10.76%)	9656(21.62%)	6239(13.97%)	2384(5.34%)
JC62	22619(50.65%)	4699(10.52%)	8690(19.46%)	6032(13.51%)	2616(5.86%)
JC63	20989(47.00%)	4907(10.99%)	9951(22.28%)	6363(14.25%)	2446(5.48%)
HY11	18738(41.96%)	5083(11.38%)	10867(24.33%)	7277(16.30%)	2691(6.03%)
HY12	18996(42.54%)	4984(11.16%)	10655(23.86%)	7202(16.13%)	2819(6.31%)
HY13	19171(42.93%)	5035(11.28%)	10684(23.93%)	7063(15.82%)	2703(6.05%)
HC11	18870(42.26%)	4871(10.91%)	10897(24.40%)	7305(16.36%)	2713(6.08%)
HC12	18850(42.21%)	5179(11.60%)	11133(24.93%)	6864(15.37%)	2630(5.89%)
HC13	19519(43.71%)	4906(10.99%)	10436(23.37%)	7099(15.90%)	2696(6.04%)
HY31	19465(43.59%)	5396(12.08%)	11331(25.37%)	5988(13.41%)	2476(5.54%)
HY32	19303(43.23%)	5238(11.73%)	11182(25.04%)	6368(14.26%)	2565(5.74%)
HY33	19188(42.97%)	5454(12.21%)	11479(25.71%)	6192(13.87%)	2343(5.25%)
HC31	19687(44.09%)	5381(12.05%)	10893(24.39%)	6272(14.05%)	2423(5.43%)
HC32	19694(44.10%)	5545(12.42%)	11120(24.90%)	5946(13.32%)	2351(5.26%)
HC33	18978(42.50%)	5448(12.20%)	11560(25.89%)	6207(13.90%)	2463(5.52%)
HY61	23682(53.03%)	5336(11.95%)	8668(19.41%)	4756(10.65%)	2214(4.96%)
HY62	23720(53.12%)	5246(11.75%)	8542(19.13%)	4860(10.88%)	2288(5.12%)
HY63	22351(50.05%)	5454(12.21%)	9351(20.94%)	5126(11.48%)	2374(5.32%)
HC61	19151(42.89%)	4989(11.17%)	10991(24.61%)	7025(15.73%)	2500(5.60%)
HC62	19030(42.61%)	5303(11.88%)	11228(25.14%)	6666(14.93%)	2429(5.44%)
HC63	18916(42.36%)	5205(11.66%)	11337(25.39%)	6727(15.06%)	2471(5.53%)

“H” represent “Hongyang”; “J” represent “Jinyan”; “Y” represent “with inoculated *B. dothidea*”; “C” represent “without inoculated *B. dothidea*”; The first number represent day of infection, the second number represent number of replications.
