# Supplementary material for: Transcriptome analysis of Actinidia chinensis in response to Botryosphaeria dothidea infection
Source: PLoS One. 2020 Jan 8;15(1):e0227303. doi: 10.1371/journal.pone.0227303 (PMC6948751; doi:10.1371/journal.pone.0227303)
Supplement: Attachment — Submitted filename: PONE-D-19-15208_reviewer-1.pdf [file pone.0227303.s013.pdf]

PLOS ONE

Transcriptome analysis of Actinidia chinensis in reponse to Botryosphaeria dothidea infection

--Manuscript Draft--

**Manuscript Number:**	PONE-D-19-15208
**Article Type:**	Research Article
**Full Title:**	Transcriptome analysis of Actinidia chinensis in reponse to Botryosphaeria dothidea infection
**Short Title:**	Transcriptome analysis of Actinidia chinensis in reponse to Botryosphaeria dothidea infection
**Corresponding Author:**	Junxi Jiang<br>Jiangxi Agricultural University<br>CHINA
**Keywords:**	ripe rot, Botryosphaeria dothidea, transcriptome, resistance , Actinidia chinensis
**Abstract:**	Ripe rot of kiwifruit, caused by Botryosphaeria dothidea, extensively damages kiwifruit prouduction. Our previous study has shown that kiwifruit variety "Jinyan" is resistant to B. dothidea and "Hongyang" is susceptible. For a comparative analysis of the response to B. dothidea infection in "Jinyan" and "Hongyang" varieties, we performed a transcriptome profiling by RNA-seq. A total of 305.24 Gb of clean bases were generated from 36 libraries of which 175.76 Gb came from the resistant variety and 129.48 Gb from the susceptible variety. From the 36 libraries generated, we identified 44,656 genes including 39,041 reference genes, 5,615 novel transcripts, and 13,898 differentially expressed genes (DEGs). Among these, 2,373 potentially defense-related genes linked to calcium signaling, mitogen-activated protein kinase (MAPK), cell wall modification, phytoalexin synthesis, transcription factors, pattern-recognition receptors, and pathogenesis-related proteins may regulate kiwifruit resistance to B. dothidea. DEGs involved in calcium signaling, MAPK, and cell wall modification in the resistant variety were induced at an earlier stage and at higher levels compared with the susceptible variety. Thirty DEGs involved in plant defense response were strongly induced in the resistant variety at all three time points. This study allowed the first comprehensive understanding of transcriptome of kiwifruit in response to B. dothidea, and may contribute to identify key genes required for disease resistance to ripe rot of kiwifruit.
**Order of Authors:**	yuanxiu wang
	junxi jiang
	Guihong Xiong
	Zhe He
	Mingfeng Yan
	Manfei Zhou
**Additional Information:**	
**Question**	**Response**
**Financial Disclosure**<br><br>Enter a financial disclosure statement that describes the sources of funding for the work included in this submission. Review the submission guidelines for detailed requirements. View published research articles from *PLOS ONE* for specific examples.	This study was supported by the National Natural Science Foundation of China (Grant No. 31460452) and Jiangxi Provincial Science and Technology Plan Project (Grant No. 20181ACF60017)

Powered by Editorial Manager® and ProduXion Manager® from Aries Systems Corporation

This statement is required for submission and **will appear in the published article** if the submission is accepted. Please make sure it is accurate.<br><br>**Unfunded studies**<br>Enter: *The author(s) received no specific funding for this work.*<br><br>**Funded studies**<br>Enter a statement with the following details:<br>• Initials of the authors who received each award<br>• Grant numbers awarded to each author<br>• The full name of each funder<br>• URL of each funder website<br>• Did the sponsors or funders play any role in the study design, data collection and analysis, decision to publish, or preparation of the manuscript?<br>• **NO** - Include this sentence at the end of your statement: *The funders had no role in study design, data collection and analysis, decision to publish, or preparation of the manuscript.*<br>• **YES** - Specify the role(s) played.<br><br>* typeset	
**Competing Interests**<br><br>Use the instructions below to enter a competing interest statement for this submission. On behalf of all authors, disclose any competing interests that could be perceived to bias this work—acknowledging all financial support and any other relevant financial or non-financial competing interests.<br><br>This statement **will appear in the published article** if the submission is accepted. Please make sure it is accurate. View published research articles from *PLOS ONE* for specific examples.	The authors have declared that no competing interests exist.

**NO authors have competing interests**<br><br>Enter: *The authors have declared that no competing interests exist.*<br><br>**Authors with competing interests**<br><br>Enter competing interest details beginning with this statement:<br><br>*I have read the journal's policy and the authors of this manuscript have the following competing interests: [insert competing interests here]*<br><br>* typeset	
**Ethics Statement**<br><br>Enter an ethics statement for this submission. This statement is required if the study involved:<br><br>• Human participants<br>• Human specimens or tissue<br>• Vertebrate animals or cephalopods<br>• Vertebrate embryos or tissues<br>• Field research<br><br>Write "N/A" if the submission does not require an ethics statement.<br><br>General guidance is provided below. Consult the submission guidelines for detailed instructions. **Make sure that all information entered here is included in the Methods section of the manuscript.**	field research

**Format for specific study types**<br><br>**Human Subject Research (involving human participants and/or tissue)**<br>• Give the name of the institutional review board or ethics committee that approved the study<br>• Include the approval number and/or a statement indicating approval of this research<br>• Indicate the form of consent obtained (written/oral) or the reason that consent was not obtained (e.g. the data were analyzed anonymously)<br><br>**Animal Research (involving vertebrate animals, embryos or tissues)**<br>• Provide the name of the Institutional Animal Care and Use Committee (IACUC) or other relevant ethics board that reviewed the study protocol, and indicate whether they approved this research or granted a formal waiver of ethical approval<br>• Include an approval number if one was obtained<br>• If the study involved *non-human primates*, add *additional details* about animal welfare and steps taken to ameliorate suffering<br>• If anesthesia, euthanasia, or any kind of animal sacrifice is part of the study, include briefly which substances and/or methods were applied<br><br>**Field Research**<br><br>Include the following details if this study involves the collection of plant, animal, or other materials from a natural setting:<br>• Field permit number<br>• Name of the institution or relevant body that granted permission	
**Data Availability**<br><br>Authors are required to make all data underlying the findings described fully available, without restriction, and from the time of publication. PLOS allows rare exceptions to address legal and ethical concerns. See the PLOS Data Policy and FAQ for detailed information.	Yes - all data are fully available without restriction

A Data Availability Statement describing where the data can be found is required at submission. Your answers to this question constitute the Data Availability Statement and **will be published in the article**, if accepted.<br><br>**Important:** Stating 'data available on request from the author' is not sufficient. If your data are only available upon request, select 'No' for the first question and explain your exceptional situation in the text box.<br><br>Do the authors confirm that all data underlying the findings described in their manuscript are fully available without restriction?	
**Describe where the data may be found in full sentences. If you are copying our sample text, replace any instances of XXX with the appropriate details.**<br><br>• If the data are **held or will be held in a public repository**, include URLs, accession numbers or DOIs. If this information will only be available after acceptance, indicate this by ticking the box below. For example: *All XXX files are available from the XXX database (accession number(s) XXX, XXX.).*<br>• If the data are all contained **within the manuscript and/or Supporting Information files**, enter the following: *All relevant data are within the manuscript and its Supporting Information files.*<br>• If neither of these applies but you are able to provide **details of access elsewhere**, with or without limitations, please do so. For example:<br><br>*Data cannot be shared publicly because of [XXX]. Data are available from the XXX Institutional Data Access / Ethics Committee (contact via XXX) for researchers who meet the criteria for access to confidential data.*<br><br>*The data underlying the results presented in the study are available from (include the name of the third party*	All relevant data are within the manuscript and its Supporting Information files.

and contact information or URL).<br>• This text is appropriate if the data are owned by a third party and authors do not have permission to share the data.<br><br>* typeset	
Additional data availability information:	

Manuscript Click here to download Manuscript renamed_3b40f.docx

Transcriptome analysis of *Actinidia chinensis* in reponse to *Botryosphaeria dothidea* infection

Yuanxiu Wang[1,2], Guihong Xiong[1], Zhe He[1], Minfeng Yan[1], Manfei Zhou[1], Junxi Jiang[1*]

1. College of Agronomy, Jiangxi Agricultural University, Nanchang, China

2. College of Bioscience and Bioengineering, Jiangxi Agricultural University Nanchang, China

* Corresponding author

E-mail: jxau2011@126.com(JJ)

Abstract

Ripe rot of kiwifruit, caused by *Botryosphaeria dothidea*, extensively damages kiwifruit prouduction. Our previous study has shown that kiwifruit variety “Jinyan” is resistant to *B. dothidea* and “Hongyang” is susceptible. For a comparative analysis of the response to *B. dothidea* infection in “Jinyan” and “Hongyang” varieties, we performed a transcriptome profiling by RNA-seq. A total of 305.24 Gb of clean bases were generated from 36 libraries of which 175.76 Gb came from the resistant variety and 129.48 Gb from the susceptible variety. From the 36 libraries generated, we identified 44,656 genes including 39,041 reference genes, 5,615 novel transcripts, and 13,898 differentially expressed genes (DEGs). Among these, 2,373 potentially defense-related genes linked to calcium signaling, mitogen-activated protein kinase (MAPK), cell wall modification, phytoalexin synthesis, transcription factors, pattern-recognition receptors, and pathogenesis-related proteins may regulate kiwifruit resistance to *B. dothidea*. DEGs involved in calcium signaling, MAPK, and cell wall modification in the resistant variety were induced at an earlier stage and at higher levels compared with the susceptible variety. Thirty DEGs involved in plant defense response were strongly induced in the resistant variety at all three time points. This study allowed the first comprehensive understanding of transcriptome of kiwifruit in response to *B. dothidea*, and may contribute to identify key genes required for disease resistance to ripe rot of kiwifruit.

Keywords: *ripe rot, Botryosphaeria dothidea*, transcriptome, resistance， A*ctinidia chinensis*

Introduction

Kiwifruit (*Actinidia chinensis*) is an economically important fruit crop grown in China New Zealand Italy and many other countries [1]. Kiwifruit ripe rot (simply known as ripe rot), caused by *Botryosphaeria dothidea* is presently one of the most important diseases in China and abroad which severely restricts the sustainable development of kiwifruit industry [2-4]. When diseases is severe, it can cause a 80% loss in kiwifruit production during the 2011-2012 in Fengxin County of Jiangxi Province, China [5]. *B. dothidea* is a dominant species of the genus with a worldwide distribution and has been reported to have a wide range of hosts. It causes dieback, branch cankers, and fruit rot in hosts including apples, pears, pistachios, and blueberries [6-9]. Fruit infections occur mostly at the early fruiting stage. However, symptoms on fruit appear from near maturity to storage, resulting in fruit drop and postharvest decay [2]. As *B. dothidea* is capable of infecting a large number of plant species and has latent infection features, developing kiwifruit varieties resistant to ripe rot through conventional breeding and biotechnology is considered to be one of the most effective management strategies.

Studies on the molecular mechanisms of resistance to kiwifruit ripe rot are limited, furthermore, works on viewing the interactivity between *B. dothidea* and other host are still few and only preliminary. Suhua Bai and his colleagues applied real-time quantitative PCR (RT-qPCR) and SDS-PAGE methods to identify a PR-4 gene from *Malus domestica*, which was involved in the defense response against *B. dothidea* infection by directly inhibiting hyphal growth [10]. Zhang et al. used infected apple branch barks to clone pathogenicity-associated Md*CNGC1* gene with RT-qPCR

examined gene expression, reporting that the expression of *MdCNGC1* gene was different among apple varieties with different resistance to *B. dothidea*. Moreover, overexpression of *MdCNGC1* in *N. benthamiana* reduced salicylic acid (SA) accumulation and callose deposition [11]. Bai et al. applied both RT-qPCR and SDS-PAGE methods, and obtained a *MdXEGIP1* gene encoding xyloglucan-specific endo-(1-4)-beta-D-glucanase inhibitor protein highly expressed in *Malus domestica* in response to *B. dothidea* infection [12]. However, these reports do not made a systematic exposition on the defense mechanism to fungal pathogens infection.

High-throughput RNA sequencing (RNA-seq) technology is a powerful and efficient method for transcriptome analysis that provides higher coverage and greater resolution of the transcriptome. Researchers use RNA-seq to quantify, profile, and discover RNA transcripts. RNA-seq has been widely used in biological and medical research. Transcriptomics has been used to study host-pathogen interactions including studies in banana and *Fusarium oxysporum f. sp. cubense* [13], maize and *Sporisorium reilianum f. sp. zeae* [14], pea and *Aphanomyces euteiches* [15], and cotton and *Verticillium dahlia* [16]. Thus, using RNA-seq to obtain transcriptome expression profiles of kiwifruit after *B. dothidea* inoculation may reveal the mechanism of interaction between *B. dothidea* and kiwifruit.

In the present study, we explored the defense response of a susceptible kiwifruit genotype (“Hongyang”, HY) and a resistant genotype (“Jinyan”, JY) infected by *B. dothidea* using RNA-seq. The differential gene expression patterns in control and inoculated fruits at three different time points were analyzed by bioinformatics. The

results of this study will offer detail of how kiwifruit responds to *B. dothidea* stress and provide new theoretical bases for developing disease resistant variety by genetic engineering.

Materials and methods

Plant materials and pathogen

Two kiwifruit (*Actinidia chinensis* Planch) varieties, *B. dothidea*-susceptible “Hongyang” (HY) and -resistant “Jinyan” (JY) (S1 Fig), used in the study were grown in Shankou kiwifruit orchard in Fengxin county of Jiangxi Province. The pathogen *B. dothidea* strain (GF27) isolated from kiwifruit was maintained in 20% glycerol (-80 ℃) at the College of Agronomy, Jiangxi Agricultural University (Jiangxi, China).

Treatments

B. dothidea strain GF27 was cultured on fresh potato dextrose agar medium at 27 ℃ for 3 days and mycelial discs of 5 mm in diameter were punched out for inoculation. Kiwifruits hanging on the tree were surface sterilized with 75% ethanol, peels were air-dried, and epidermal tissue of 5 mm in diameter was removed. This was then inoculated with a mycelial disc of 5 mm diameter and covered with moist cotton. In parallel, control fruits were inoculated with sterile agar discs. All treated and control fruits were covered with plastic bags to maintain humidity. Three biological replicates were maintained for each treatment, three fruits were pooled together for each replicate. Based on our previous observations on the stages of infection, we sampled control and treated fruits of the resistant and susceptible varieties at 1, 3, and 6 days after inoculation for transcriptome analysis. The flesh surrounding the discs were collected,

frozen in liquid nitrogen, transported to the laboratory on dry ice, and stored at -80 ℃.

RNA isolation, library construction, and sequencing

Total RNA was isolated from frozen kiwifruits using TRIzol reagent (Invitrogen, CA, USA) following manufacturer's instructions. RNA degradation and contamination was monitored on 1% agarose gels and RNA integrity was assessed using the RNA Nano 6000 Assay Kit with the Agilent bioanalyzer 2100 (Agilent Technologies, CA, USA). The purity was assessed using a NanoPhotomere® UV-Vis spectrophotometer (Implen, CA, USA) and concentration was estimated using Qubit® RNA Assay Kit in Qubit® 2.0 Flurometer (Life Technologies, CA, USA).

Nuclear RNA (3μg per sample) was used and 36 cDNA libraries were generated using NEBNext® UltraTM RNA Library Prep Kit for Illumina ® (NEB, USA) according to manufacturer's protocol. The mRNA was purified from total RNA using oligo(dT) magnetic beads and cleaved into small fragments. First- and second-strand cDNA were synthesized using random hexamer primer and M-MuLV reverse transcriptase (RNase H-), followed by DNA polymerase I and RNase H. After adenylation of the 3' ends of the cDNA fragments, NEBNext adapter oligonucleotides were ligated to prepare for hybridization. The library fragments were purified with AMPure XP system (Beckman Coulter, Beverly, USA) to select cDNA fragments of preferentially 150–200 bp in length. The required fragments were enriched by PCR amplification using Phusion high-fidelity DNA polymerase, universal PCR primers, and index (X) primer. PCR products were purified (AMPure XP system), and the library quality was assessed on the Agilent Bioanalyzer 2100 system.

Clustering of the index-coded samples was performed on a cBot cluster generation system using TruSeq PE Cluster Kit v3-cBot-HS (Illumina) according to manufacturer's instructions. The library preparations were sequenced on an Illumina HiSeq platform to generate 125 bp/150 bp paired-end reads.

Mapping and differential expression analysis of RNA-seq data

Raw reads were preprocessed to remove adapter sequences, reads containing ploy-N, and low-quality sequences (Q<20). Clean reads were mapped to the kiwifruit reference genome (ftp://bioinfo.bti.cornell.edu/pub/kiwifruit) using TopHat (2.0.12) [17]. Novel transcripts were identified from TopHat alignment results by Cufflinks [18]. The reads per gene were counted using HTSeqv0.6.1(https://pypi.python.org/pypi/HTSeq) [19] and normalized to FPKM (fragments per kilobase of exon model per million mapped reads) [20]. Differentially expressed genes (DEGs) between the two samples was performed using the DESeq R package (1.10.1) [21]. The resulting *P*-values were adjusted using the Benjamini and Hochberg [22] method to control the false discovery rate. Genes with an adjusted *P*-value <0.05 found by DESeq were defined as significant DEGs.

Functional analysis of differentially expressed genes

Gene Ontology (GO) enrichment analysis of differentially expressed genes was implemented using GOseq R package [23] in which gene length bias was corrected. GO terms with corrected *P*-values < 0.05 were considered significantly enriched. Kyoto

Encyclopedia of Genes and Genomes (KEGG; http://www.genome.jp/kegg/) pathway analysis of differentially expressed genes was performed by KOBAS software [24]. KEGG pathway with corrected *P*-values ≤ 0.05 were considered significantly enriched by DEGs.

Quantitative real-time PCR analysis of differentially expressed genes

To test the reliability of RNA-seq data, eight coexpressed genes from resistant varieties were selected for qRT-PCR. Specific primers were designed using Primer 5.0 software (PREMIER Biosoft, Palo Alto, CA, USA) and synthesized by Sangon (Shanghai, China). Primer pairs are listed in S1 Table. Actin isform B (ACTB) [25] served as the internal reference gene. The cDNA was synthesized from 1 μg of total RNA using GoScriptTM Reverse Transcription System (Promega, Madison, USA) in 20 μL of reaction mixture. The qRT-PCR reactions were performed using GoTaq® qPCR master mix (Promega, Madison, USA) on a Bio-Rad CFX 96 real time PCR system (Bio-Rad, Hercules, CA, USA). The total reaction volume was 20 μL including 0.5 μL F/R primer, 2.0 μL template, and 10 μL master mix. Reaction conditions were as follows: 3 min denaturation at 95 ℃, followed by 42 cycles of 95 ℃ for 30 s and 60 ℃ for 30 s. Following amplification, melting curve analysis was performed by increasing the temperature from 65 ℃ to 95 ℃ (0.5 ℃/5 s) to confirm the specificity of the PCR amplification. Three replicates with three technical repeats per experiment were maintained for each gene. A $2^{-\Delta\Delta CT}$ algorithm was applied for quantitative gene expression analysis [26].

Results

Illumina sequencing and mapping to the reference genome

To generate the transcriptome profile of kiwifruit during *B. dothidea* infection, samples were collected from control and inoculated resistant and susceptible varieties at 1, 3, and 6 days after infection. Thirty six cDNA libraries were prepared and sequenced using Illumina HiSeq 2000 system. Transcriptome analyses of 12 samples including JY1, JY3, JY6, JC1, JC3, JC6, HY1, HY3, HY6, HC1, HC3, and HC6 (JY and HY indicate inoculated, JC and HC indicate non-inoculated control; each sample had three biological replicates) were performed.

A total of 1,219,912,954 raw reads were generated from the 36 libraries, with 305.24 Gb high-quality (Q＞20)clean bases selected for further analysis. More than 41 million reads were obtained for each library, 52.20%–86.26% of the total mapped reads were aligned onto the kiwifruit reference genome. Among which, 1.50%–2.46% of reads were mapped to multiple locations, 50.71%–83.98% were mapped to single locations, (Table 1, S2 Fig). The number of mapped reads did not show significant difference from the mapped chromosomes (S3 Fig).

All read counts were normalized to fragments per kilobase of exon per million fragments mapped (FPKM) to obtain the relative levels of expression. As shown in

Table 1. Summary of sequencing data quality and the statistics of the transcriptomes assembly.

Sample name	Raw reads	Clean reads	clean bases （G）	Total mapped	Multiple mapped	Uniquely mapped
JY11	58,402,262	56,137,592	8.42	37,162,992 (66.20%)	1,087,417 (1.94%)	36,075,575 (64.26%)

JY12	65,083,550	62,579,642	9.39	42,132,349 (67.33%)	1,172,726 (1.87%)	40,959,623 (65.45%)
JY13	60,566,738	57,909,694	8.69	36,797,803 (63.54%)	941,027 (1.62%)	35,856,776 (61.92%)
JC11	71,430,208	68,397,674	10.26	44,788,294 (65.48%)	1,338,086 (1.96%)	43,450,208 (63.53%)
JC12	67,086,948	64,400,654	9.66	43,041,396 (66.83%)	1,292,735 (2.01%)	41,748,661 (64.83%)
JC13	73,680,888	70,670,374	10.6	46,430,338 (65.70%)	1,246,164 (1.76%)	45,184,174 (63.94%)
JY31	78,391,904	75,088,168	11.26	48,868,871 (65.08%)	1,370,529 (1.83%)	47,498,342 (63.26%)
JY32	64,091,754	61,529,028	9.23	40,122,764 (65.21%)	1,114,367 (1.81%)	39,008,397 (63.40%)
JY33	66,754,456	63,921,842	9.59	40,954,653 (64.07%)	1,158,414 (1.81%)	39,796,239 (62.26%)
JC31	71,153,290	68,388,676	10.26	45,439,208 (66.44%)	1,244,652 (1.82%)	44,194,556 (64.62%)
JC32	65,257,352	62,809,534	9.42	41,646,147 (66.31%)	1,235,654 (1.97%)	40,410,493 (64.34%)
JC33	63,352,396	60,983,688	9.15	40,251,570 (66%)	1,109,449 (1.82%)	39,142,121 (64.18%)
JY61	63,871,012	61,498,706	9.22	35,093,030 (57.06%)	1,152,941 (1.87%)	33,940,089 (55.19%)
JY62	68,345,924	65,738,986	9.86	43,651,442 (66.40%)	1,408,734 (2.14%)	42,242,708 (64.26%)
JY63	74,633,558	71,819,850	10.77	45,504,616 (63.36%)	1,346,213 (1.87%)	44,158,403 (61.48%)
JC61	69,540,170	66,708,904	10.01	43,627,321 (65.40%)	1,191,540 (1.79%)	42,435,781 (63.61%)
JC62	67,538,660	64,986,180	9.75	40,680,640 (62.60%)	1,241,799 (1.91%)	39,438,841 (60.69%)
JC63	70,731,884	68,117,950	10.22	44,992,629 (66.05%)	1,242,771 (1.82%)	43,749,858 (64.23%)
HY11	46,155,756	44,550,560	6.68	35,457,999 (79.59%)	1,095,502 (2.46%)	34,362,497 (77.13%)
HY12	44,008,054	42,314,410	6.35	33,620,186 (79.45%)	919,524 (2.17%)	32,700,662 (77.28%)
HY13	53,597,000	48,154,334	7.22	40,947,696 (85.03%)	1,066,608 (2.21%)	39,881,088 (82.82%)
HC11	52,234,306	41,177,902	6.18	33,805,189 (82.10%)	882,293 (2.14%)	32,922,896 (79.95%)

HC12	60,739,060	48,858,294	7.33	40,257,899 (82.40%)	1,095,755 (2.24%)	39,162,144 (80.15%)
HC13	57,923,842	47,488,480	7.12	40,962,841 (86.26%)	1,079,853 (2.27%)	39,882,988 (83.98%)
HY31	48,231,106	46,774,344	7.02	36,998,612 (79.10%)	1,010,305 (2.16%)	35,988,307 (76.94%)
HY32	47,009,114	45,890,956	6.88	36,442,434 (79.41%)	1,092,762 (2.38%)	35,349,672 (77.03%)
HY33	66,566,704	63,079,362	9.46	50,161,651 (79.52%)	1,547,783 (2.45%)	48,613,868 (77.07%)
HC31	54,408,376	42,946,688	6.44	35,293,763 (82.18%)	955,872 (2.23%)	34,337,891 (79.95%)
HC32	51,895,392	41,005,136	6.15	33,416,139 (81.49%)	981,411 (2.39%)	32,434,728 (79.10%)
HC33	57,337,234	45,315,078	6.8	37,367,117 (82.46%)	997,012 (2.20%)	36,370,105 (80.26%)
HY61	61,454,572	59,636,020	8.95	31,132,736 (52.20%)	893,298 (1.50%)	30,239,438 (50.71%)
HY62	51,127,280	49,614,034	7.44	27,425,469 (55.28%)	758,879 (1.53%)	26,666,590 (53.75%)
HY63	55,927,700	54,279,534	8.14	32,479,616 (59.84%)	884,160 (1.63%)	31,595,456 (58.21%)
HC61	46,020,478	44,045,818	6.61	34,562,191 (78.47%)	953,468 (2.16%)	33,608,723 (76.30%)
HC62	50,566,214	48,997,936	7.35	39,242,480 (80.09%)	1,007,203 (2.06%)	38,235,277 (78.03%)
HC63	50,923,392	49,034,168	7.36	38,662,471 (78.85%)	1,028,902 (2.10%)	37,633,569 (76.75%)

HY11, HY12, and HY13: HY with inoculated *B.dothidea* cultured for 1 day

HC11, HC12, and HC13: HY without inoculated *B.dothidea* cultured for 1 day

HY31, HY32, and HY33: HY with inoculated *B.dothidea* cultured for 3 days

HC31, HC32, and HC33: HY without inoculated *B.dothidea* cultured for 3 days

HY61, HY62, and HY63: HY with inoculated *B.dothidea* cultured for 6 days

HC61, HC62, and HC63: HY without inoculated *B.dothidea* cultured for 6 days

JY11, JY12, and JY13: JY with inoculated *B.dothidea* cultured for 1 day

JC11, JC12, and JC13: JY without inoculated *B.dothidea* cultured for 1 day

JY31, JY32, and JY33: JY with inoculated *B.dothidea* cultured for 3 days

JC31, JC32, and JC33: JY without inoculated *B.dothidea* cultured for 3 days

JY61, JY62, and JY63: JY with inoculated *B.dothidea* cultured for 6 days

JC61, JC62, and JC63: JY without inoculated *B.dothidea* cultured for 6 days

Figs 1A and 1B, all 36 samples displayed similar FPKM distribution and similar FPKM density distribution. About 50% of the total number of genes (44,656) had FPKM ≥ 1 and approximately 5% of the genes had FPKM ≥ 60 in each library (S2 Table). The high correlation coefficient of the three biological replicates assured that the analysis was reliable (Fig 1C).

Fig 1. Bioinformatic analysis of RNA-seq data. (A) FKPM distribution for each sample. (B) FPKM density distribution for each sample. (C) Pearson correlation between 36 sets of kiwifruit samples.

Analysis of differentially expressed genes in response to *B. dothidea*

A total of 13,898 DEGs were detected in "Jinyan" (resistant, R) and "Hongyang" (susceptible, S) samples (S3 Table). In R, 579 (352 up-regulated; 227 down-regulated), 4421 (2,680 up-regulated; 1741 down-regulated), and 574 (538 up-regulated; 36 down-regulated) DEGs were detected in the pairwise comparisons JY1 vs JC1, JY3 vs JC3,

and JY6 vs JC6. Up-regulated genes were more in number than down-regulated genes at all three stages of infection (Fig 2A, S4 Fig). In S, 803 (639 up-regulated; 164 down-regulated), 1,109 (937 up-regulated; 172 down-regulated), and 11,998 (5,724 up-regulated; 6,274 down-regulated) DEGs were detected in the pairwise comparisons HY1 vs HC1, HY3 vs HC3, and HY6 vs HC6 (Fig 2B, S4 Fig). Up-regulated genes were more in number than down-regulated genes at the first and the second time point (the 1st and 3rd day after infection), while up-regulated genes were less in number compared to down-regulated genes at the third time point (the 6th day after inoculation). We detected 48 DEGs in R and 207 DEGs in S with sustained expression at different time points of the same variety (Figs 2A and 2B). However, no sustained differentially expressed genes were found in both varieties at different time points. Interestingly, 36 DEGs in R with sustained expression were detected in S at the third time point (S3 Table).

Hierarchical clustering of all DEGs was done based on the log10 (FPKM +1) for 36 samples (Fig 2C). The expression of DEGs in R and S before and after *B. dothidea* infection was different, suggesting that R and S varieties had specific response to *B. dothidea*. The inoculated samples JY3 and HY6 had the similar expression patterns, indicating that the timing of induced gene expression was earlier in R than in S.

Fig 2. Venn diagram of differentially expressed genes of kiwifruit during interaction with *B. dothidea*. (A) Venn diagram of differentially co-expressed genes of “Jinyan” (JY, resistant) at different time points after *B. dothidea* inoculation. (B) Venn diagram of differentially co-

expressed genes of “Hongyang” (HY, susceptible) at different time points after *B. dothidea* inoculation. (C) Heat map of DEGs across three infection stages in both cultivars. Expression values of all libraries are presented as log10 (FPKM +1).

Functional classification of resistant and susceptible kiwifruit transcriptome

We performed GO term enrichment analysis ($P<0.05$) to identify the functions of DEGs in each pairwise comparison at three time points in two varieties. Through the analysis of the up-regulated DEGs, we observed significantly enriched terms in three major GO categories such as biological process, molecular functions, and cellular component (S4 Table). Four GO terms associated with oxidation-reduction were found (GO: 0055114, GO: 0016491, GO: 0051213, GO: 0016706) at three time points in S of which GO: 0055114 and GO: 0016491 were exclusively present on 6th day in R. GO: 0005976, GO: 0016762, and GO: 0048046 were correlated with the changes in cell wall and were found in JY3 vs JC3 and JY6 vs JC6 pairwise comparisons, whereas no GO terms were enriched in JY1 vs JC1. The results showed that defense responses were different in resistant and susceptible varieties after infection. This finding allowed us to consider cell wall as a major player in kiwifruit in providing resistance against *B. dothidea*. Concurrently, we analyzed the GO terms of the down-regulated DEGs in the inoculated samples of R and S varieties (S4 Table). There was no GO term associated with sustained down-regulated genes at the three time points in R or S. Some GO terms (S4 Table; GO: 0016762 and GO: 0048046) up-regulated in the R variety were down-regulated in the S variety. Their difference in expression indicated a potential role in

regulating the resistance mechanism to *B. dothidea* in R.

To further identify the biological pathways in which the DEGs were involved, we performed KEGG analysis. In total, 6,557 DEGs were assigned to 113 KEGG pathways and 35 of them were significantly enriched (P-value$<$0.05). The “metabolic pathway” (223, 1.61 %) was the most significant enriched term, followed by “protein processing” (136, 0.98%) and “carbon metabolism” (124, 0. 89 %) (S5 Table). Comparing treated samples with control samples in both varieties, 24 pathways were significantly up-regulated and 11 pathways were significantly down-regulated (S5 Table). KEGG pathway analysis failed to enrich in down- regulated pathway in HY1 vs HC1, and JY1 vs JC1. Not unexpectedly, plant-pathogen interaction (ath04626) was up-regulated in R species at three time points (1st, 3rd, 6th day after incubation), while this pathway was up-regulated only in HY3 vs HC3. Phenylpropanoid biosynthesis (ath00940) DEGs, which have a positive role in plant resistance response, were exclusively down-regulated in HY3 vs HC3. These results indicated that defense response was more activ[illegible] the R variety than in the S variety. Notably, photosynthesis (ath00195), photosynthesis-antenna proteins (ath00196), porphyrin and chlorophyll metabolism (ath00860), and carbon fixation in photosynthetic organisms (ath00710) were the critical down-regulated pathways in JY3 vs JC3. This observation is consistent with a previous study that proved the role of photosynthesis-associated pathways in the resistance mechanism [27].

Expression analysis of defense-related genes and screening of candidate genes in response to *B. dothidea*

In order to investigate the defense mechanism of kiwifruits against *B. dothidea* infection, we identified 2,377 potential defense-related genes by searching the keywords in the gene annotation by referring to the literature on defense response. These defense-related genes included 519 PRRs, 32 MAPK, 583 TFs, 83 resistance proteins (R Proteins), 105 pathogenesis-related protein (PRP), 217 calcium signaling coding genes, 312 cell wall modification-related genes, and 523 hormone metabolism (S6 Table). Comparing R genotype and S genotype, these DEGs involved in resistance to *B. dothidea* showed distinct expression patterns. In the R genotype, similar expression patterns were found at the first time point and at the third time point. Moreover, few down-regulated DEGs appeared at the third time point and the up-regulated DEGs were more than down-regulated DEGs at the second time point. In the S genotype, a similar number of DEGs were identified at the first time point and at the second time point. Although the number of up-regulated genes increased at the third time point, the number of down-regulated genes was not much lower (Fig 3). Collectively, 30 suatained up-regulated genes encoding PRRs, MAPK, calcium signaling, TFs, hormone metabolism, and cell wall modification-related genes were identified to play role in kiwifruit resistance to *B. dothidea* (Fig 4).

Fig 3. Expression pattern of DEGs encoding defense-related genes in both cultivars at different time points after *B. dothidea* inoculation. The horizontal axis indicates days post inoculation and the vertical axis indicates the number of differentially expressed defense-related genes of the transcriptome. Orange bars indicate down-regulated DEGs of HY; Green bars

indicate up-regulated DEGs of HY; Blue bars indicate down-regulated DEGs of JY; and Purple bars indicate up-regulated DEGs of JY.

Fig 4. Level of expression of thirty defense-related genes of both cultivars at different time points after *B.dothidea* inoculation. a indicates DEGs from the MAPK cascades category; b from PRRs category; c from Ca^{2+} influx category; d from TFs category; e from cell wall modification category; f from hormone category; and g from other defense systems. Y-axis shows the fold changes between both cultivars, the positive and negative values represent up-regulated and down-regulated respectively.

Validation of DEGs by quantitative real-time PCR analysis

To confirm the accuracy of RNA-seq data, eight co-expressed DEGs involved in resistant to *B. dothidea* in R were selected for RT-qPCR analysis (Achn251121, Achn104901, Achn040411, Achn012851, Achn386421, Achn372801, Achn315051, Achn327381). Analysis showed the same expression of up-regulation or down-regulation as RNA-seq (Fig 5), however, the degree of expression varies between the two data sets beacause of the different sensitivity of Illumina sequencing and RT-qPCR. In general, these results suggested the reliability of RNA-seq to analyse the transcriptome of resistant and susceptible plants during pathogen infection.

Fig 5. qRT- PCR validation of eight candidate genes identified by RNA-seq at 1, 3, and 6 days in “HY” and “JY” cultivars. Left vertical axis represents relative gene expression levels determined by qRT- PCR, right verticalaxis represents RNA-seq fold change in differentially

expressed genes, horizontal axis represents days post inoculation. Asterisks indicate significant data meet (P-value <0.05). Each experiment had three technical replicates and error bars represent standard deviation (n = 3).

Discussion

As far as we know, this is the first report to use RNA-seq technology to identify the genes of resistant and susceptible kiwifruit varieties in response to *B. dothidea* invasion. A total of 13,898 DEGs were detected between "Jinyan" and "Hongyang" and 2,373 potential defense-related genes involved in PTI and ETI were identified by searching the keywords in the gene annotation, determined by a manual literature search (S6 Table) and are discussed below.

Pathogen perception by pattern-recognition receptors

In the present study, there was no significant difference in the expression levels of PRRs genes in R genotype compared with in S genotype at the first time point (1st day after incubation), however, the expression levels in R were higher than in S (S6 Table; Fig 4). Two PRR genes (Achn240671, Achn325051) were sustained up-regulated at three time points in R genotype. In S genotype, Achn240671 was up-regulated at the first and the third time point, Achn325051 was only up-regulated at the third time point (S6 Table; Fig 4). First layer of innate immune system in plants is based on a sensitive perception of pathogen or microbe-associated molecular patterns (PAMPs) through pattern recognition receptors (PRRs) at the cell surface, resulting in PTI to halt further colonization [28, 29]. These DEGs which are the potential candidate genes of response to *B. dothidea* have been reported to provoke a rapid immune response in plants and is

speculated that PTI perhaps plays a role in kiwifruit resistance to *B. dothidea*. Achn278911 was sustained down-regulated at three time points in R genotype but up-regulated at the second time point (3rd day after incubation) in S genotype. For the opposite expression pattern in two varieties, the role of Achn278911 deserves to be further studied.

Activation of MAPK cascades

In the present study, some DEGs encoding MAPK showed distinct expression patterns between two varieties. Compared with the susceptible, mitogen-activated protein kinase (MAPK) cascades were induced early and almost up-regulated at all three time points in the R (Fig 3). MAPK cascades are universal signaling modules in eukaryotes and respond to environmental stresses like cold, drought, and salinity [30]. They play crucial roles in signaling plant immune response including phytoalexin biosynthesis, stomatal closure, and hypersensitive response (HR) [30, 31]. Three co-expressed genes (Achn091221, Achn315051, and Achn315061) were up-regulated in R. Achn315051 and Achn315061 encoding MAPK3/MAPK6 and Achn343991 (S6 Table, Fig 4) encoding a kiwifruit anthranilate N-benzoyltransferase protein involved in the biosynthesis of phytoalexin showed sustained up-regulation in R. MAPK cascades were activated following the activation of WRKY-type transcription in Arabidopsis during *Botrytis cinerea* infection and regulated phytoalexin production[32]. Taken together, our analysis suggests that MAPK cascades may regulate the biosynthesis of phytoalexin in response to *B. dothidea* infection.

Calcium signaling

Calcium is a ubiquitous intracellular messenger, which regulates plant responses to abiotic and biotic stresses like heat, drought, salt, and pathogens [33]. Stimulus induces increase in intracellular Ca^{2+} ions that combine with Ca^{2+}-binding proteins, leading to physiological and biochemical response to pathogen invasion [34, 35]. Recent research has shown that Ca^{2+} signaling is necessary for stomatal closure and can activate cell wall modification following oligogalacturonides (OGs) perception which were petic fragments of plant cell walls [36, 37]. In the current study, Achn304251 (CaM), Novel00884 (CDPK), and Achn199221 (CaMBP) were up-regulated in R genotype but were down-regulated in S genotype (S6 Table). Three transcripts encoding CaM or CML (Achn089411, Achn201941, and Achn327381), two transcripts encoding CaMBPs (Achn012851 and Achn124411), and two transcripts encoding other Ca^{2+}-binding proteins (Achn017121 and Achn024491) (S6 Table, Fig 4) showed sustained, up-regulated expression in R but not in S. These up-regulated DEGs encoding Ca^{2+}/CaM-binding proteins indicate the role of calcium signaling pathway in defense response such as stomatal closure, phytoalexin biosynthesis, and accumulation of PR protein. Six genes associated with cell wall modification (Achn367751, Novel00171, Achn291611, Novel05351, Achn386421, and Achn299241) (S6 Table, Fig 4) exhibited up-regulation at all three time points in R, which implies that Ca^{2+} might participate in cell wall modification in plant defense.

Transcription factors (TFs)

Transcription factors (TFs) play direct or indirect roles in different cell signaling pathways, and regulate plant defense processes [38]. In this study, WRKY, GATA and

PLATZ TFs were proven to be involved in coping with pathogens. 22 DEGs encoding WRKY TFs were almost up-regulated only in R by second and third time points following inoculation with *B. dothidea*. This was in agreement with the previous observations in *Arabidopsis* that WRKY is required for resistance to necrotrophic fungal pathogens [39]. In our study, 5 DEGs encoding GATAs TFs were all up-regulated in R, 14 DEGs encoding GATA TFs were induced in S, 8 of which were down-regulated. Additionally, two GATA genes (Achn166151 and Achn294851) (S6 Table, Fig 4) were sustained up-regulated in R. In spite of only 2 DEGs encoding PLATZ TFs were induced in R, one PLATZ gene (Achn020891) was sustained down-regulated in R.

PLATZ and GATA were all plant-specific zinc-dependent DNA-binding protein [40], there are no available reports to prove that GATA and PLATZ play roles in defense responses to pathogens. [illegible]viously, the roles of GATA in low temperature-induced stress responses [41], chlorophyll biosynthesis, and glutamate synthase in Arabidopsis [42] have been elucidated. GmPLATZ1 gene was specifically induced by drought, high salinity, or abscisic acid (ABA) in soybean [43]. Achn166151 and Achn294851 were sustained up-regulated and Achn020891 was sustained down-regulated in R, indicating that they may be involved in the resistance interaction between kiwifruit and *B. dothidea*.

Plant hormone signal transduction

In order to resist a wide range of microbial pathogens, plants produce various hormones including abscisic acid (ABA), salicylic acid (SA), jasmonic acid (JA), ethylene (ET),

auxin, and brassinosteroids (BRs) that regulate physiological processes at low concentrations [44]. In the present study, SA and JA were not significantly different in R and S varieties that indicate that they do not play a role in defense reaction.

ABA is considered as a negative regulator of plant defense responses against pathogens [45]. However, recent research suggests that the phytohormone ABA enhances plant immunity by regulating stomatal closure and callose deposition [44]. Protein phosphatase (PP2C) is often considered as a negative regulatory factor in the ABA signal transduction pathway [46]. On the contrary, some reports indicated PP2C acts as a positive regulator to activate ABA signal transduction pathway to promote stomatal closure and enhance plant immunity [47, 48].

In this study, expression of PP2C genes in R occurred earlier than those in S, five PP2C DEGs (Achn286741, Novel04947, Achn194931, Achn104901, and Achn251121) were detected in the study and showed different expression patterns in both varieties. Achn286741 and Novel04947 were up-regulated in R but down-regulated in S; Achn194931, Achn104901, and Achn251121 were sustained up-regulated at all three time points in R and only at the third time point in S (S6 Table, Fig 4). Collectively, our data suggest that the expression of ABA signaling-related genes varieties were significantly different in the two varieties, which indicates that it plays a significant role in the response to *B. dothidea* infection. Combining with identified the up-regulated Ca^{2+}, TFs and MAPK DEGs in the current study, we speculated that ABA may activate Ca^{2+} regulation and MAPK cascades to regulate stomatal closure, which is consistent with the report on the core regulatory network in ABA responses [48]. Three ABA

signaling-related genes (Achn194931, Achn104901, and Achn251121) may be involved in the process of stomatal closure to confine the infection and spread of *B. dothidea*.

Consistent with ABA, auxin signaling is also antagonistic to SA signaling and promote plant susceptibility to the pathogen [49]. However, several research showed that auxin signaling can mediate resistance to *Plectosphaerella cucumerina* and *P. cinnamomi* in *A. thaliana* [50, 51]. Consistent with previous reports, the up-regulation of auxin-related DEGs nearly doubled at the second time point in R, and the down-regulation of auxin-related DEGs doubled at 6th day in S. Two auxin-related DEGs (Achn030331 and Achn154151) were up-regulated in R but down-regulated in S during *B. dothidea* infection. The expression of Achn040411 encoding auxin-independent growth promoter increased continuously only in R (S6 Table, Fig 4).

Cyclin-dependent kinase inhibitor (ICK) regulates correct cell cycle progression and effects on growth in transgenic plants [52]. In this study, only 4 DEGs were induced in both varieties, which demonstrated sustained up-regulation of an ICK (Achn266081) in R (S6 Table, Fig 4). Currently, Safae Hamdoun demonstrated the positive role of ICK gene in regulating innate immunity in *Arabidopsis thaliana* resistance to *Pseudomonas syringae* [53]. Present study was consist with the previous report, but the exact role of Achn266081 still need further study.

Cell wall-mediated defense response

Plant cell wall is composed of cellulose, hemicellulose, pectin, and a few structural proteins [54]. It serves as a defense barrier that protects the plant from pathogen

penetration. Miedes et al. reported that the reduction in expression of *XTH* facilitates *Penicillium expansum* to infect tomato [55]. *KCS* gene is involved in very long chain fatty acid (VLCFA) biosynthesis which was proven to play a role in pathogen resistance [56, 57]. Study by Wei et al demonstrated that the *Arabidopsis thaliana* transgenic overexpressing the extensin gene limits *Pseudomonas syringae* invasiveness [58]. Similarly, the mutation in *bc1* encoding a COBRA protein caused cell wall thinness and reduction in cellulose content in rice [59]. We detected that five DEGs (S6 Table, Fig 4) involved in cell wall modification including Xyloglucan endotransglucosylase /hydrolase (*XTHs*) genes (Achn367751 and Novel00171), 3-ketoacyl-CoA synthase (*KCS*) gene (Achn291611), Extensin gene (Novel05351), *COBRA* gene (Achn386421) were sustained up-regulation in R compared with S. Consistent with these previous reports, our results indicate that the up-regulation of five DEGs (Achn367751, Novel00171, Achn291611, Novel05351, and Achn386421) might prevent *B. dothidea* infection by thickening cuticular wax, cross-linking, and strengthening plant cell walls. Cellulose synthase (*CesA*) gene encodes cellulose enzymes involved in biosynthesis of cell wall components, it was up-regulated in watermelon during defense against *Fusarium oxysporum f. sp. niveum* [60]. *CesA* genes were down-regulated in the incompatible interaction of *myb46 Arabidopsis* with *Botrytis cinerea* [61]. It is worth noting that two cellulose synthase genes Achn299241 sustained up-regulation and Achn343521 sustained down-regulation were identified in the current study. These results imply that cellulose synthase may regulate plant defense response by synthesizing cellulose to reinforce cell wall or by inhibiting cellulose synthesis to

activate novel defense pathways in response to *B. dothidea*.

An earlier work showed that drastic *GDSL1* featured with a conserved GDSL motif at N' terminus and encoding a member of the esterase/lipase protein silencing leads to reduction in tomato cuticle thickness and cutin monomer content [62]. It was also reported that *Arabidopsis* GLIP1-elicited systemic resistance to *Alternaria brassicicola* is dependent on ethylene signaling [63]. In our study, Achn372801 encoding GDSL sustained up-regulation at all three time points in R. However, no DEGs involved in ET signaling pathways exhibited sustained up-regulation in R. Our analysis suggests that Achn372801 assists resistance to *B. dothidea* by thickening cuticular wax or disrupting fungal spore integrity in cooperation with signaling of other hormones.

Overall, the mechanism of plant defense response against *B. dothidea* is complex. Expression of transcripts was detected early in R than in S and the tendency of genes up-regulated was obvious in R than in S at the third time point. These may indicate the resistance specificity and early timing of the resistant variety.

Conclusions

In this study, we performed a transcriptome analysis to reveal the defense response of resistant and susceptible varieties of kiwifruit during *B. dothidea* infection. A total of 305.24 Gb clean bases were generated and 13,898 DEGs were detected in 36 libraries of kiwifruits. A total of 2,373 potential defense-related genes were identified; The DEGs involved in PRRs, MAPK signaling, calcium signaling, Hormone metabolism pathways, TFs pathways and cell wall modification ,which were reported previously as relevant to defense response, were explored, and 30 candidate genes relate to plant

defense response were identified from these pathways for future study. We propose a putative network underlying the sustained expression of defense-related DEGs in "Jinyan" (Fig 6). PRR proteins were activated by some effector proteins, leading to activation of MAPK signaling or calcium signaling. Hormone metabolism and TFs pathways were also activated, which trigger defense responses like cell wall modification, stomatal closure, and phytoalexin generation. This study provides a better understanding of the molecular basis of defense against *B. dothidea* in kiwifruit and would facilitate improvement in disease control genetic engineering.

Fig 6. Schematic representation of the response in resistant cultivar to *B. dothidea* infection. Dotted lines represent the phytoalexin biosynthesis and signaling pathway in response to *B. dothidea*. Dashed lines represent the cell wall-associated defense responses in kiwifruit. Solid lines represent ABA-dependent defense pathways in response to *B. dothidea*.

Acknowledgments

This study was supported by the National Natural Science Foundation of China (Grant No. 31460452) and Jiangxi Provincial Science and Technology Plan Project (Grant No. 20181ACF60017)

References

1. Everett KR, Taylor RK, Romberg MK, Rees-George J, Fullerton RA, Vanneste JL, et al. First report of *Pseudomonas syringae pv. actinidiae* causing kiwifruit bacterial canker in New Zealand. Australasian Plant Disease Notes. 2011; 6(1): 67-71. https://doi.org/10.1007/s13314-011-0023-9.
2. Li C, Jiang JX, Leng JH, Li BM ,Yu Q, Tu GQ. Isolation and identification of pathogenic fungi causing

fruit rot of kiwifruit in fengxin county. Acta Agriculturae Universitatis Jiangxiensis. 2012; 34(2): 259-263.

3. Koh YJ, Hur JS, Jung JS. Postharvest fruit rots of kiwifruit (*Actinidia deliciosa*) in Korea. New Zealand Journal of Crop and Horticultural Science. 2005; 33(3): 303-310. https:// doi.org/10.1080/01140671. 2 005.9514363.

4. Pennycook SR, Samuels GJ. *Botryosphaeria* and *Fusicoccum* species associated with ripe fruit rot of *Actinidia deliciosa* (Kiwifruit) in New Zealand. Mycotaxon. 1985; 24: 445-458.

5. Li C, Wang L, Jiang JX, Wang YX, Zhao SG, Li BM,et al. Biological characteristics of the main pathogenic fungi causing fruit ripe rot of kiwifruit and disease resistance of kiwifruit cultivars. Acta Agriculturae Universitatis Jiangxiensis. 2014; 36(5): 1061-1065. https://doi.org/10.13836/ j.jjau. 201416 9.

6. Jurick WM, Ii, Vico I, Gaskins VL, Janisiewicz WJ, Peter KA. First report of *Botryosphaeria dothidea* causing white rot on apple fruit in Maryland. Plant Disease. 2013; 97(7): 999. https://doi.org/10.1094/ PDIS-01-13-0053-PDN PMID: 30722551

7. Garibaldi A, Bertetti D, Poli A, Gullino ML. First report of fruit rot in pear caused by *Botryosphaeria dothidea* in Italy. Plant Disease. 2012; 96(6): 910. https://doi.org/10.1094/PDIS-02-12-0130-PDN PMID: 30727390

8. Wunderlich N, Costa SS, Tpoi RP, Ash GJ. First report of *Botryosphaeria dothidea* causing shoot blight and cankers of pistachio in Australia. Australasian Plant Disease Notes. 2012; 7(1): 47-49. https:// doi.org/10.1007/s13314-012-0045-y

9. Choi IY. First report of bark dieback on blueberry caused by *Botryosphaeria dothidea* in Korea. Plant Disease. 2011; 95(2): 227. https://doi.org/10.1094/PDIS-05-10-0371 PMID:30743439

10. Bai SH, Dong CH, Li BH, Dai HY. A PR-4 gene identified from Malus domestica is involved in the defense responses against *Botryosphaeria dothidea*. Plant Physiol Biochem. 2013; 62(1): 23-32. https:// doi.org/10.1016/j.plaphy.2012.10.016 PMID:23178481

11. Zhang WW, Dong CH, Zhang YG, Zhu J, Dai HY, Bai SH. An apple cyclic nucleotide-gated ion channel gene highly responsive to *Botryosphaeria dothidea* infection enhances the susceptibility of *Nicotiana benthamiana* to bacterial and fungal pathogens. Plant Science. 2018; 269: 94-105. https://doi.org/10.10 16/j.plantsci.2018.01.009 PMID:29606221

12. Bai SH, Dong CH, Zhu J, Zhang YG, Dai HY. Identification of a xyloglucan-specific endo-(1-4)-beta- d -glucanase inhibitor protein from apple (*Malus × domestica Borkh*.) as a potential defense gene against *Botryosphaeria dothidea*. Plant Science. 2015; 231: 11-9. https://doi.org/10.1016/j.plantsci.2014.11.00 3 PMID: 25575987

13. Li CY, Deng GM, Yang J, Viljoen A, Jin Y, Kuang RB, et al. Transcriptome profiling of resistant and susceptible Cavendish banana roots following inoculation with *Fusarium oxysporum f. sp. cubense* tropical race 4. BMC Genomics. 2012; 13(1): 374. https://doi.org/10.1186/1471-2164-13-374 PMID: 22863187

14. Zhang SP, Xiao YN, Zhao JR, Wang FG, Zheng YL. Digital gene expression analysis of early root infection resistance to *Sporisorium reilianum f. sp. zeae* in maize. Molecular Genetics and Genomics. 2013; 288(1-2): 21-37. https://doi.org/10.1007/s00438-012-0727-3 PMID: 23196693

15. Hosseini S, Elfstrand M, Heyman F, Dan FJ, Karlsson M. Deciphering common and specific transcriptional immune responses in pea towards the oomycete pathogens *Aphanomyces euteiches and Phytophthora pisi*. BMC Genomics. 2015; 16(1): 1-18. https://doi.org/ 10.1186/s12864-015-1829-1 PMID: 26293353

16. Xu L, Zhu LF, Tu LL, Liu LL, Yuan DJ, Jin L, et al. Lignin metabolism has a central role in the resistance

of cotton to the wilt fungus *Verticillium dahliae* as revealed by RNA-Seq-dependent transcriptional analysis and histochemistry. Journal of Experimental Botany. 2011; 62(15): 5607-5621. https://doi.org/10.1093/jxb/err245 PMID: 21862479

17. Trapnell C, Pachter L, Salzberg SL. TopHat: discovering splice junctions with RNA-Seq. Bioinformatics. 2009; 25(9):1105-1111. https://doi.org/ 10.1093/bioinformatics/btp120 PMID: 19289445

18. Trapnell C, Roberts A, Goff L, Pertea G, Kim D, Kelley DR, et al. Differential gene and transcript expression analysis of RNA-seq experiments with TopHat and Cufflinks. Nat Protoc. 2012; 7(3): 562-578. https://doi.org/10.1038/nprot.2012.016 PMID: 22383036

19. Anders S, Pyl PT, Huber W. HTSeq--a Python framework to work with high-throughput sequencing data. Bioinformatics. 2015; 31(2): 166-169. https://doi.org/ 10.1093/bioinformatics/btu638 PMID: 25260700

20. Trapnell C, Williams BA, Pertea G, Mortazavi A, Kwan G, van Baren MJ, et al. Transcript assembly and quantification by RNA-Seq reveals unannotated transcripts and isoform switching during cell differentiation. Nature Biotechnology. 2010; 28(5): 511-5. https://doi.org/10.1038/nbt.1621 PMID: 20436464

21. Robinson MD, Oshlack A. A scaling normalization method for differential expression analysis of RNA-seq data. Genome Biology. 2010; 11(3): R25. https://doi.org/10.1186/gb-2010-11-3-r25 PMID: 20196867

22. Benjamini Y, Hochberg Y. Controlling the false discovery rate - a practical and powerful approach to multiple testing. Journal of the Royal Statistical Society. 1995; 57(1): 289-300. https://doi.org/ 10.2307/2346101

23. Young MD , Wakefield MJ , Smyth GK , Oshlack A. Gene ontology analysis for RNA-seq: accounting for selection bias. Genome biology. 2010, 11(2): R14. https://doi.org/10.1186/gb-2010-11-2-r14 PMID: 20132535

24. Mao X, Cai T, Olyarchuk JG, Wei L. Automated genome annotation and pathway identification using the KEGG Orthology (KO) as a controlled vocabulary. Bioinformatics. 2005; 21(19): 3787-93. https://doi.org/ 10.1093/bioinformatics/bti430 PMID: 15817693.

25. Jain M, Nijhawan A, Tyagi AK, Khurana JP. Validation of housekeeping genes as internal control for studying gene expression in rice by quantitative real-time PCR. Biochemical and Biophysical Research Communications. 2006; 345(2): 646-651. https://doi.org/10.1016/j.bbrc.2006.04.140 PMID: 16690022

26. Livak KJ, Schmittgen TD. Analysis of relative gene expression data using real-time quantitative PCR and the $2^{-\Delta\Delta CT}$ method. Methods. 2001; 25(4): 402-408. https://doi.org/10.1006/meth.2001.1262. PMID: 11846609.

27. Kangasjarvi S, Neukermans J, Li SC, Aro EM, Noctor G. Photosynthesis, photorespiration, and light signalling in defence responses. Journal of Experimental Botany. 2012; 63(4): 1619-1636. https://doi.org/10.1093/jxb/err402 PMID: 22282535

28. Jones JD, Dangl JL. The plant immune system. Nature. 2006; 444(7117): 323-329. https://doi.org/10.1038/nature05286 PMID: 17108957.

29. Kishimoto K, Kouzai Y, Kaku H, Shibuya N, Minami E, Nishizawa Y. Perception of the chitin oligosaccharides contributes to disease resistance to blast fungus *Magnaporthe oryzae* in rice. The Plant Journal. 2010; 64(2): 343-354. https://doi.org/10.1111/j.1365-313X.2010.04328.x PMID: 21070413.

30. Pathak RK, Taj G, Pandey D, Kumar A, Arora S. Modeling of the MAPK machinery activation in response to various abiotic and biotic stresses in plants by a system biology approach. Bioinformation.

2013; 9(9): 443-449. https://doi.org/10.6026/97320630009443 PMID: 23847397

31. Meng XZ, Zhang SQ. MAPK cascades in plant disease resistance signaling. Annual Review of Phytopathology. 2013; 51(1): 245-266. https://doi.org/10.1146/annurev-phyto-082712-102314 PMID: 23663002

32. Ren DT, Liu YD, Yang Kwang-Y, Han L, Mao GH, Glazebrook J, et al. A fungal-responsive MAPK cascade regulates phytoalexin biosynthesis in *Arabidopsis*. Proceedings of the National Academy of Sciences. 2008; 105(14): 5638-5643. https://doi.org/10.1073/pnas.0711301105 PMID: 18378893

33. Dodd AN, Kudla J, Sanders D. The language of calcium signaling. Annual Review of Plant Biology. 2010; 61(1): 593-620. https://doi.org/10.1146/annurev-arplant-070109-104628 PMID: 20192754

34. Lecourieux D, Ranjeva R, Pugin A. Calcium in plant defence-signalling pathways. New Phytologist. 2006; 171(2): 249-69. https://doi.org/10.1111/j.1469-8137 PMID: 16866934

35. Reddy VS, Ali GS, Reddy ASN. Characterization of a pathogen-induced calmodulin-binding protein: mapping of four Ca^{2+}-dependent calmodulin-binding domains. Plant Molecular Biology. 2003; 52(1): 143-159. https://doi.org/10.1023/a:1023993713849 PMID: 12825696

36. Allen GJ, Chu SP, Schumacher K, Shimazaki CT, Vafeados D, Kemper A, et al. Alteration of stimulus-specific guard cell calcium oscillations and stomatal closing in *Arabidopsis det3* mutant. Science. 2000; 289(5488): 2338-2342. https://doi.org/10.1126/science.289.5488.2338 PMID: 11009417

37. Moscatiello R, Mariani PD, Sanders D, Maathuis FJM. Transcriptional analysis of calcium-dependent and calcium-independent signalling pathways induced by oligogalacturonides. Journal of Experimental Botany. 2006; 57(11): 2847-2865. https://doi.org/10.1093/jxb/erl043 PMID: 16868046

38. Eulgen T. Regulation of the *Arabidopsis* defense transcriptome. Trends in Plant Science. 2005; 10(2): 71-78. https://doi.org/10.1016/j.tplants.2004.12.006 PMID: 15708344

39. Zheng ZY, Qamar SA, Chen ZX, Mengiste T. Arabidopsis WRKY33 transcription factor is required for resistance to necrotrophic fungal pathogens. Plant Journal. 2010; 48(4): 592-605. https://doi.org/ 10.11 11/j.1365-313x.2006.02901.x PMID: 17059405

40. Nagano Y, Furuhashi H, Inaba T, Sasaki Y. A novel class of plant-specific zinc-dependent DNA-binding protein that binds to A/T-rich DNA sequences. Nucleic Acids Research. 2001; 29(20): 4097-4105. https://doi.org/10.1093/nar/29.20.4097 PMID: 11600698

41. Bonthala VS, Mayes K, Moreton J, Blythe M, Wright V, May ST, et al. Identification of gene modules associated with low temperatures response in bambara groundnut by network-based analysis. Plos One. 2016; 11(2): e0148771. https://doi.org/10.1371/journal.pone.0148771 PMID: 26859686

42. Hudson D, Guevara D, Yaish MW, Hannam C, Long N, Clarke JD, et al. GNC and CGA1 modulate chlorophyll biosynthesis and glutamate synthase (GLU1/Fd-GOGAT) expression in *Arabidopsis*. Plos One. 2011; 6(11): e26765. https://doi.org/10.1371/journal.pone.0026765 PMID: 22102866

43. So HA, Choi SJ, Chung E, Lee JH. Molecular characterization of stress-inducible PLATZ. Plant Omics Journal. 2015; 8(6): 479-484.

44. Bari R, Jones JDG. Role of plant hormones in plant defence responses. Plant Molecular Biology. 2009; 69(4): 473-488. https://doi.org/10.1007/s11103-008-9435-0 PMID: 19083153

45. Mauch-Mani B, Mauch F. The role of abscisic acid in plant-pathogen interactions. Current Opinion in Plant Biology. 2005; 8(4): 409-414. https://doi.org/10.1016/j.pbi.2005.05.015 PMID: 15939661

46. Wang WM, Ma XF, Zhang Y, Luo MC, Wang GL, Bellizzi M, et al. PAPP2C interacts with the atypical disease resistance protein RPW8.2 and negatively regulates salicylic acid-dependent defense responses in *Arabidopsis*. Molecular Plant. 2012; 5(5): 1125-1137. https://doi.org/10.1093/mp/sss008 PMID: 2233 4594

47. Chan ZL. Expression profiling of ABA pathway transcripts indicates crosstalk between abiotic and biotic stress responses in *Arabidopsis*. Genomics. 2012; 100(2): 110-115. https://doi.org/10.1016/j.ygen o.2012.06.004 PMID: 22709556

48. Umezawa T, Nakashima K, Miyakawa T, Kuromori T, Tanokura M, Shinozaki K, et al. Molecular basis of the core regulatory network in ABA responses: sensing, signaling and transport. Plant Cell Physiology. 2010; 51(11): 1821-1839. https://doi.org/10.1093/pcp/pcq156 PMID: 20980270

49. Wang D, Pajerowska-Mukhtar K, Culler AH, Dong XN. Salicylic acid inhibits pathogen growth in plants through repression of the auxin signaling pathway. Current Biology. 2007; 17(20): 1784-1790. https://doi.org/10.1016/j.cub.2007.09.025 PMID: 17919906

50. Llorente F, Muskett P, Sanchez-Vallet A, Lopez G, Ramos B, Sanchez-Rodriguez C, et al. Repression of the auxin response pathway increases *Arabidopsis* susceptibility to necrotrophic fungi. Molecular Plant. 2008; 1(3): 496-509. https://doi.org/10.1093/mp/ssn025 PMID: 19825556

51. Eshraghi L, Anderson JP, Aryamanesh N, Mccomb JA, Shearer B, Hardy GS. Suppression of the auxin response pathway enhances susceptibility to *Phytophthora cinnamomi* while phosphite-mediated resistance stimulates the auxin signalling pathway. BMC Plant Biology. 2014; 14(1): 68. https://doi.org/ 10.1186/1471-2229-14-68 PMID: 24649892

52. Veylder LD, Beeckman T, Beemster GT, Krols L, Terras F, Landrieu I, et al. Functional analysis of cyclin-dependent kinase inhibitors of *Arabidopsis*. Plant Cell. 2001; 13(7): 1653-1667. https://doi.org/ 10.1105/tpc.010087 PMID: 11449057

53. Hamdoun S, Zhang C, Gill M, Kumar N, Churchman M, Larkin JC, et al. Differential roles of two homologous cyclin-dependent kinase inhibitor genes in regulating cell cycle and innate immunity in *Arabidopsis*. Plant Physiology. 2016; 170(1): 515-527. https://doi.org/10.1104/pp.15.01466 PMID: 265 61564

54. Cosgrove DJ. Growth of the plant cell wall. Nat Rev Mol Cell Biol. 2005; 6(11): 850-861. https://do i.org/ 10.1038/nrm1746 PMID: 16261190

55. Eva M, Lorences EP. The implication of xyloglucan endotransglucosylase/hydrolase (XTHs) in tomato fruit infection by *Penicillium expansum* Link. A. J Agric Food Chem. 2007; 55(22): 9021-9026. htt ps://doi.org/10.1021/jf0718244 PMID: 17960871

56. Todd J, Post-Beittenmiller D, Jaworski JG. KCS1 encodes a fatty acid elongase 3-ketoacyl-CoA synthase affecting wax biosynthesis in *Arabidopsis thaliana*. The Plant Journal. 2010; 17(2): 119-130. https://doi.org/10.1046/j.1365-313X.1999.00352.x PMID: 10074711

57. Raffaele S , Leger A , Roby D. Very long chain fatty acid and lipid signaling in the response of plants to pathogens. Plant Signaling & Behavior. 2009; 4(2): 94-99. https://doi.org/10.4161/psb.4.2.7580 PMID: 19649180

58. Wei G, Shirsat AH. Extensin over-expression in *Arabidopsis* limits pathogen invasiveness. Molecular Plant Pathology. 2010; 7(6): 579-592. https://doi.org/10.1111/j.1364-3703.2006.00363.x PMID: 205074 71

59. Li YH, Qian Q, Z YH, Yan MX, Sun L, Zhang M, et al. BRITTLE CULM1, which encodes a COBRA-like protein, affects the mechanical properties of rice plants. Plant Cell. 2003; 15(9): 2020-2031. https://doi .org/10.1105/tpc.011775 PMID:

60. Lü GY, Guo SG, Zhang HY, Geng LH, Song FM, Fei ZJ. Transcriptional profiling of watermelon during its incompatible interaction with *Fusarium oxysporum f. sp. niveum*. European Journal of Plant Pathology. 2011; 131(4): 585-601. https://doi.org/10.1007/s10658-011-9833-z

61. Ramírez V, García-Andrade J, Vera P. Enhanced disease resistance to *Botrytis cinerea* in myb46

Arabidopsis plants is associated to an early down-regulation of CesA genes. Plant Signaling & Behavior. 2011; 6(6): 911-913. https://doi.org/10.4161/psb.6.6.15354 PMID: 21617373

62. Girard AL, Mounet F, Lemaire-Chamley M, Gaillard C, Elmorjani K, Vivancos J, et al. Tomato GDSL1 is required for cutin deposition in the fruit cuticle. Plant Cell. 2012; 24(7): 3106-3121. https://doi.org/10.1105/tpc.112.101055 PMID: 22805434

63. Kwon SJ, Jin HC, Lee S,Nam MH, Chung JH,Kwon S, et al. GDSL lipase-like 1 regulates systemic resistance associated with ethylene signaling in *Arabidopsis*. Plant Journal for Cell & Molecular Biology. 2009; 58(2): 235-245. https://doi.org/10.1111/j.1365-313x.2008.03772.x PMID: 19077166

Author Contributions

Conceptualization: Junxi Jiang, Yuanxiu Wang

Data curation: Guihong Xiong, Yuanxiu Wang

Supervision: Manfei Zhou

Validation: Zhe He, Minfeng Yan

Writing-original draft: Yuanxiu Wang

Writing-review & editing: Yuanxiu Wang, Junxi Jiang

Supporting information

S1 Fig. Disease symptoms observed on kiwifruit 8 days after B.dothidea inoculation.

(JPG)

S2 Fig. Classification of raw reads.

（RAR）

S3 Fig. Read Density in Chromosomes.

（RAR）

S1 Table. Primers used for RT-qPCR analysis of differentially expressed genes.

(DOC)

S2 Table. Number of genes at different FPKM interval after significance level correction.

（PDF）

S3 Table. Differentially expressed genes analysis and annotation.

（XLS）

S4 Fig. Volcano plot of differentially expressed kiwifruit genes during interaction with *B.dothidea* inoculation.

(RAR)

S4 Table. Significantly enriched GO terms.

（XLS）

S5 Table. Significantly enriched KEGG terms.

（XLS）

S6 Table. Expression analysis of kiwifruit defense-related genes.

（XLS）

Figure Click here to download Figure Fig1.tif.tif

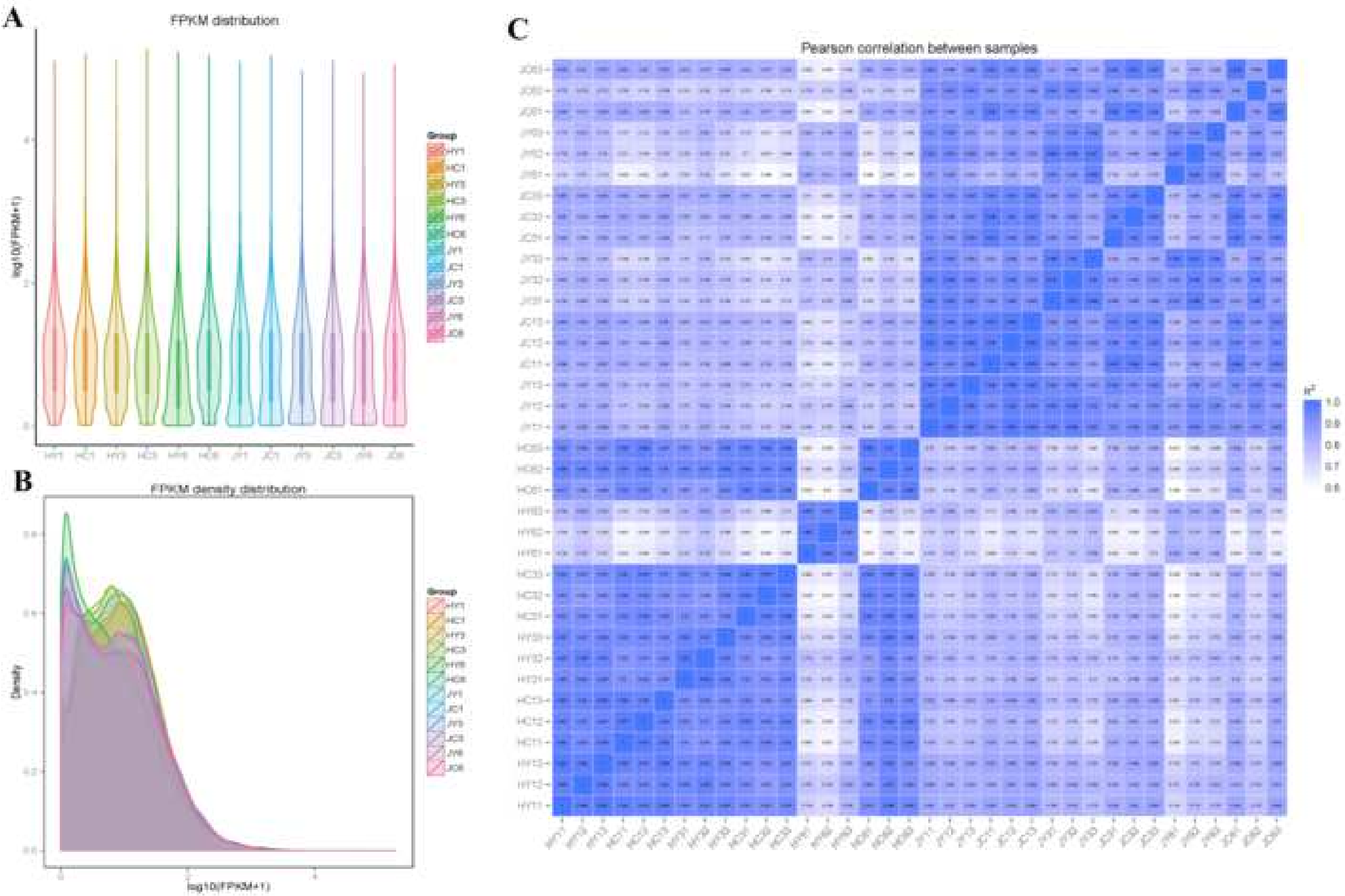

Figure

Click here to download Figure Fig2.tif.tif

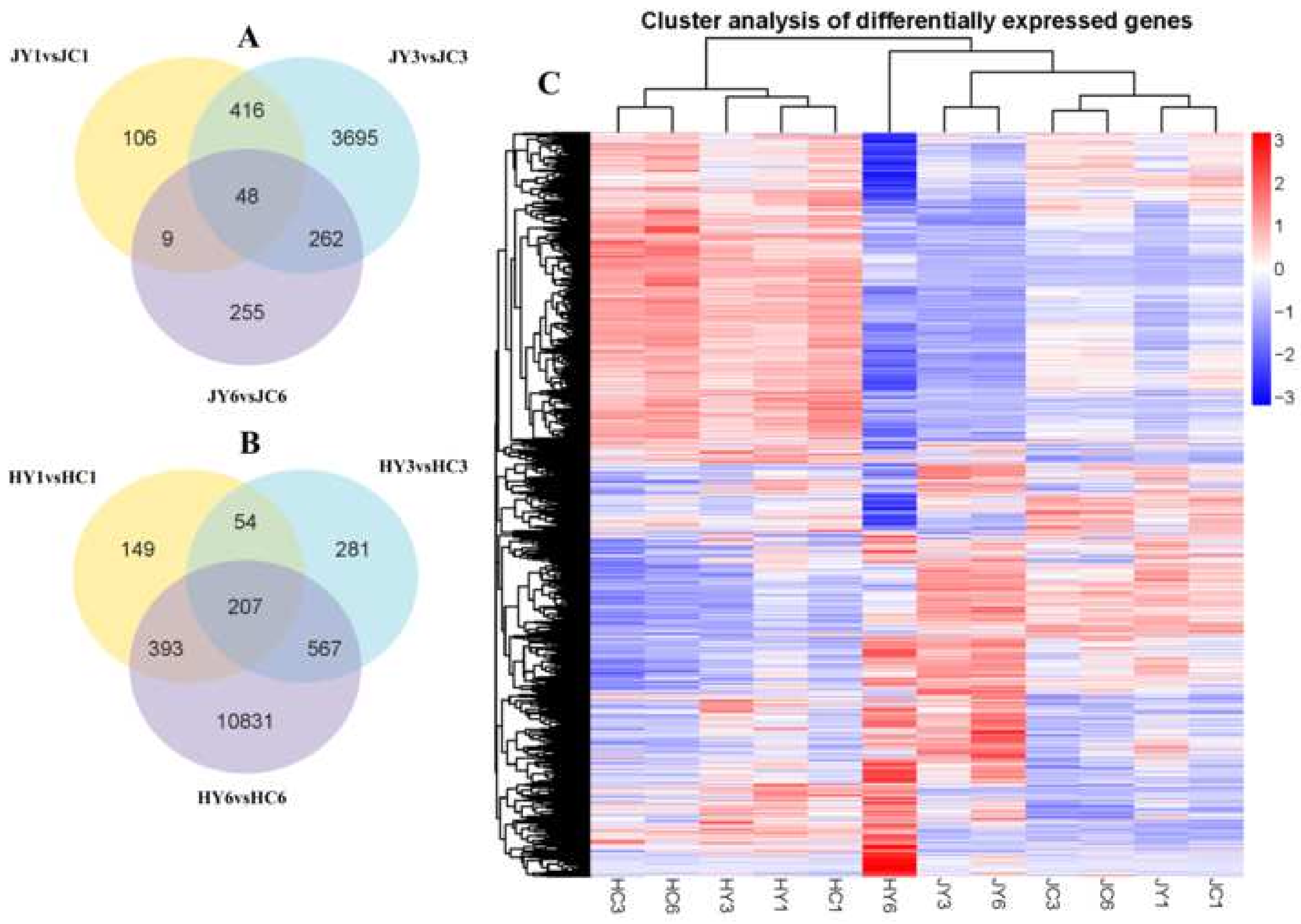

Figure

Click here to download Figure Fig3.tif.tif

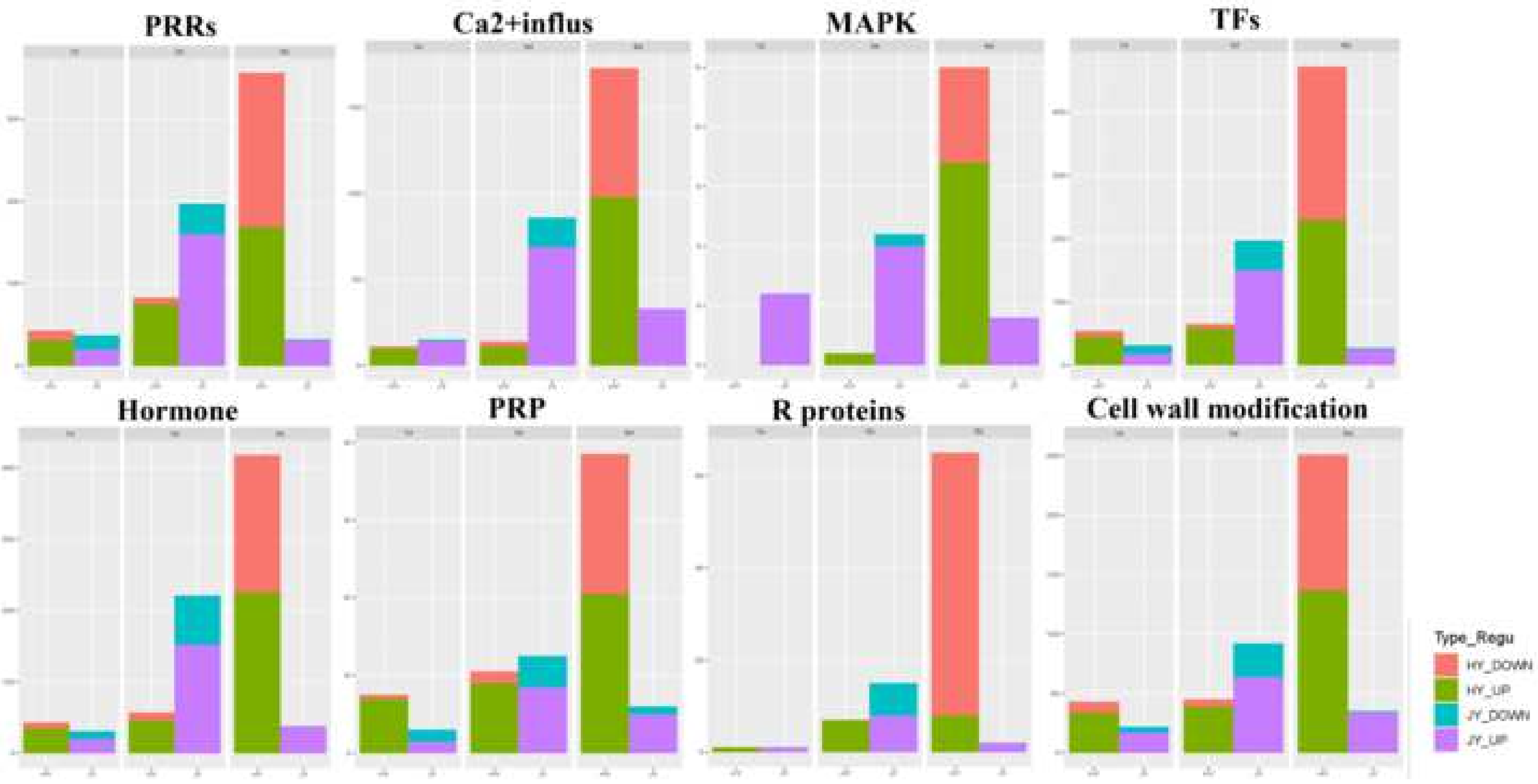

Figure Click here to download Figure Fig4.tif.tif

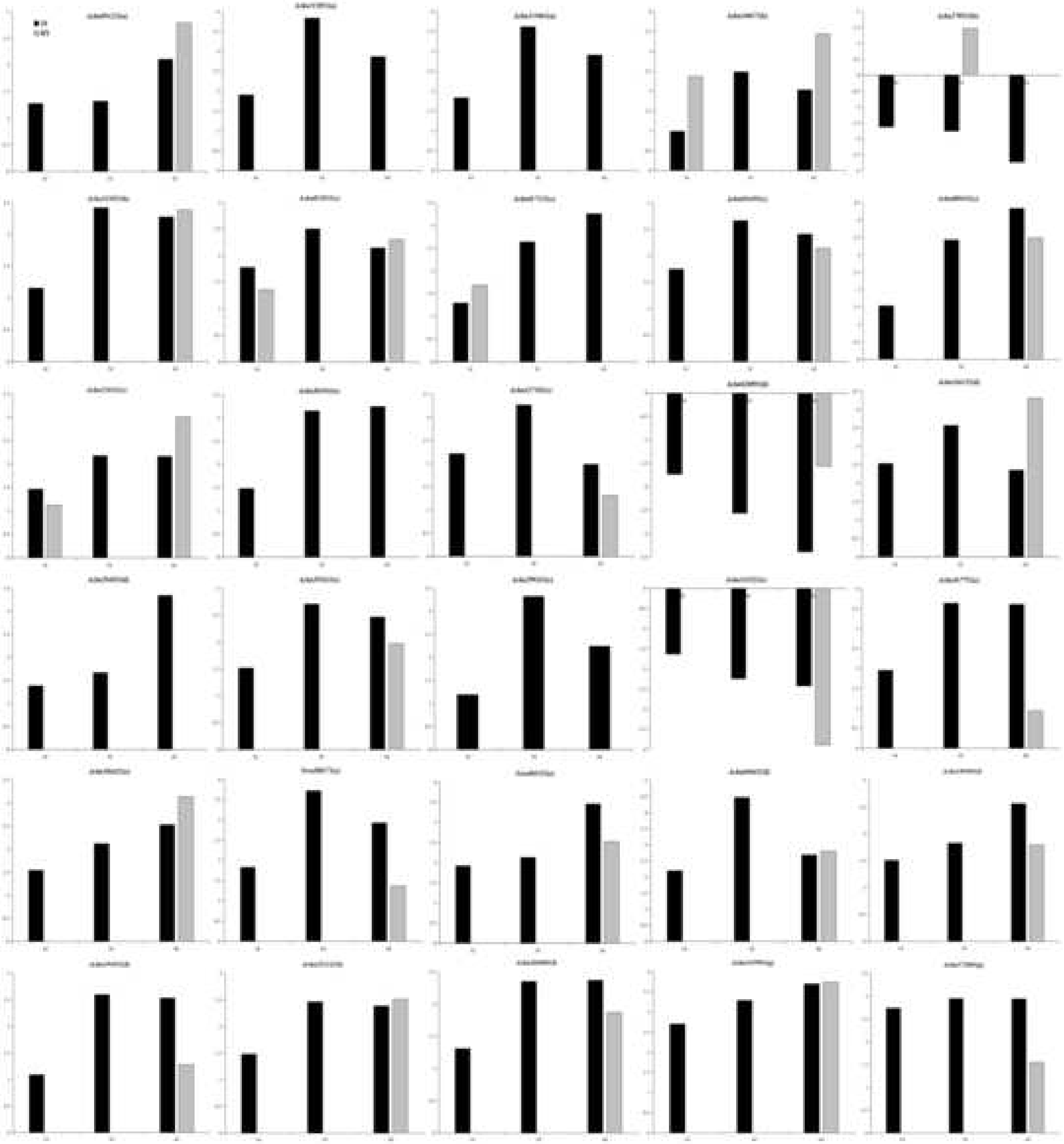

Figure

Click here to download Figure Fig5.tif.tif

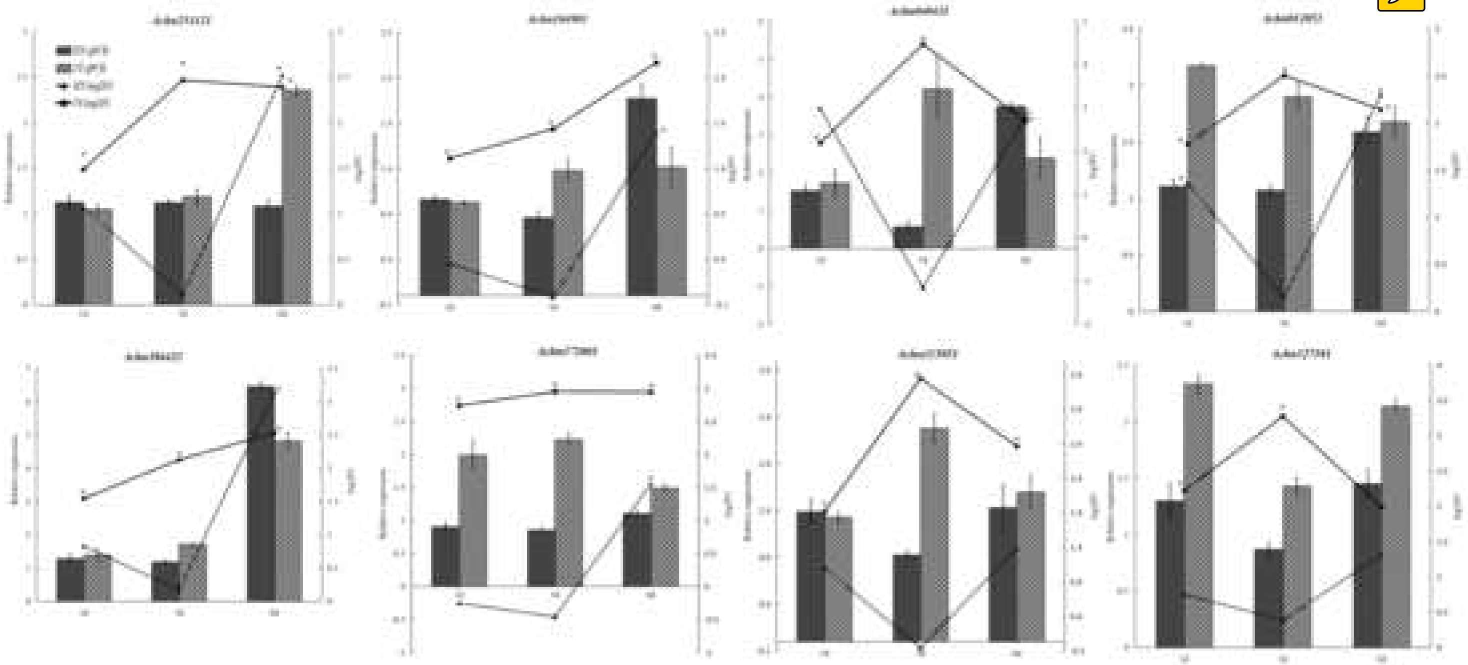

Figure

Click here to download Figure Fig6.tif.tif

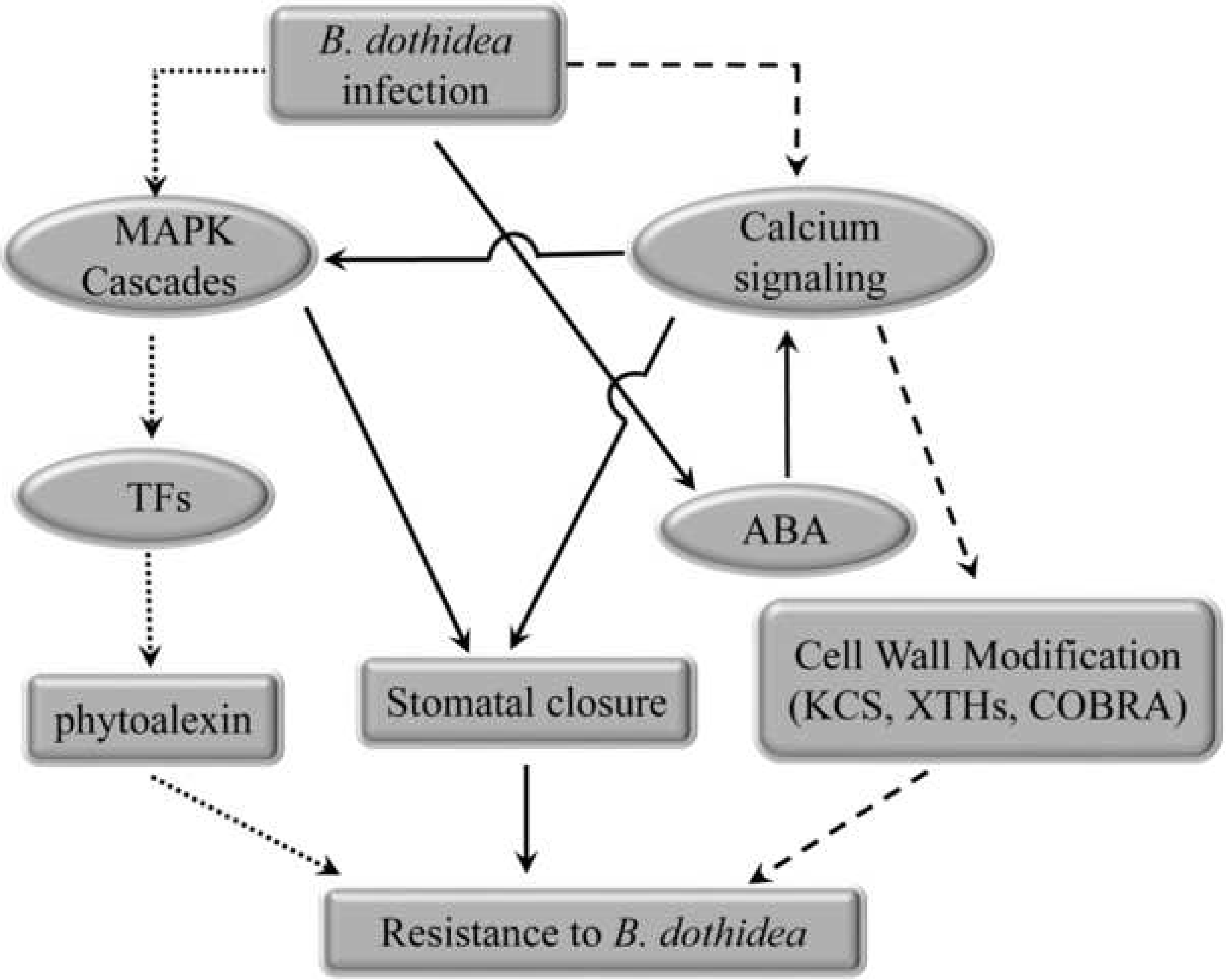

Click here to access/download
Supporting Information
S1 Fig.jpg

Click here to access/download
Supporting Information
S1 Table.docx

Click here to access/download
Supporting Information
S2 Fig.rar

Click here to access/download

Supporting Information

S2 Table.pdf

Click here to access/download
Supporting Information
S3 Fig.rar

Click here to access/download
Supporting Information
S3 Table.xls

Click here to access/download
Supporting Information
S4 Fig.rar

Click here to access/download
Supporting Information
S4 Table.xls

Click here to access/download
Supporting Information
S5 Table.xls

Click here to access/download
Supporting Information
S6 Table.xlsx
