# Supplementary material for: Transcriptome analysis of Actinidia chinensis in response to Botryosphaeria dothidea infection
Source: PLoS One. 2020 Jan 8;15(1):e0227303. doi: 10.1371/journal.pone.0227303 (PMC6948751; doi:10.1371/journal.pone.0227303)
Supplement: Attachment — Submitted filename: Response to reviewer 5.docx [file pone.0227303.s014.docx]

**Comments from Reviewer** **5**

Q1: Proper scientific nomenclature should be followed for all the organisms.

A1: Thanks, we have made changes. All changes are shown in red font in the manuscript.

Q2: Some *Actinidia chinensis* genes have a name/homolog name/function assigned to them. It should be uniform throughout the text, since the homologous genes in other annotated plants are known.

A2: Thanks for the suggestion. We made corrections. All changes are shown in red font in the manuscript.

Q3: Authors should clarify the control treatment, and the culture method for the pathogen propagule used in this study clearly in the methods section. How was the pathogen isolated from the host for further experiment, how was it cultured, and how/what stage was it harvested in 20% glycerol for storage. The authors do describe culturing the pathogen on PDA for infection but that is so generic.

A3: Thank you for your assessment. We have we revised them to “Two kiwifruit varieties, B. dothidea-susceptible “Hongyang” (HY) and -resistant “Jinyan” (JY), of Shankou kiwifruit orchard in Fengxin county of Jiangxi Province were used. HY fruits harvested at 140 days after flowering and JY fruits harvested at 180 days after flowering were selected for the experiments. A total of 210 *B. dothidea* strains were isolated from the lesions with the typical symptoms of ripe rot in the infected HY fruits. These strains were cultured at 27 ℃ for 3 days, preserved on potato dextrose agar slants, and maintained in 20% glycerol (-80 ℃) at the College of Agronomy, Jiangxi Agricultural University (Jiangxi, China). After virulence assessment, “GF27”, the highly pathogenic strain of *B. dothidea*, was selected for inoculation in Shankou kiwifruit orchard.”. *B. dothidea* strain GF27 was cultured on fresh potato dextrose agar at 27 ℃ for 3 days and mycelial discs of 5 mm in diameter were punched out for inoculation. Healthy and ripe fruits on the trees were surface sterilized with 75% ethanol, peels were allowed to air-dry, and an epidermal tissue of 5 mm in diameter was removed from each fruit. Mycelial disc of *B. dothidea* was used to inoculate each wound. Control fruits received agar discs lacking mycelium. All treated and control fruits were covered with plastic bags to maintain humidity. We sampled control and treated fruits of the resistant and susceptible varieties for transcriptome analysis at 1, 3, and 6 days after inoculation. The flesh surrounding the discs were collected, frozen in liquid nitrogen, transported to the laboratory on dry ice, and stored at -80 ℃. Flesh surrounding the discs taken from five different fruits randomly selected from three different trees were polled as a biological replicate. Three independent biological replicates were prepared for each treatment. Samples collected at three different inoculation time points (1, 3, and 6 d) for “Hongyang” (HY, with *B. dothidea* inoculation; HC, without inoculation) and “Jinyan” (JY, with *B. dothidea* inoculation; JC, without inoculation) were used to construct thirty six fruit libraries for RNA-seq, which were named as HY11, HY12, HY13, HC11, HC12, HC13, HY31, HY32, HY33, HC31, HC32, HC33, HY61, HY62, HY63, HC61, HC62, HC63, JY11, JY12, JY13, JC11,JC12, JC13, JY31, JY32, JY33, JC31, JC32, JC33, JY61, JY62, JY63, JC61, JC62, and JC63 (Page 4–5, line87–118).

Q4: The figures 1, 3, 4 and 5 are highly pixelated, without a clear label it is difficult to correlate with the observations/conclusions drawn in the manuscript text.

A4: Thanks for the suggestions. We have made corrections, as shown in figures 1, 3, 4 and 5 files (Page 13, line235–241), (Page 16–17, line305–316), (Page 17–18, line331–334).

Q5: The authors are advised to submit the gene/transcript sequences mentioned in the paper with the name of the closest homology from an annotated genome and the corresponding sequences as a FASTA file format in supplementary information. This would account for data transparency and replication for researchers in this system or in other plant-pathogen systems.

A5: Thanks for the suggestion, the relevant information has been summarized in supplementary information S7.

Q6: This is a novel study with some interesting results that would benefit researchers working in other systems. That being said, is the sequencing data from this study being made publicly available or deposited in GenBank/NCBI. If not authors should consider doing that when resubmitting the article.

A6: Thanks, we add “The raw sequencing data of this study have been deposited in the BIG Data Center GSA database (Accession No. CRA001649).” (Page 7, line 144–146).

We tried our best to improve the manuscript and made some changes in the manuscript. These changes did not influence the content or framework of the paper. All reference formats in the text have been revised. In addition, we did not list all changes here, but they are marked in red in the revised paper.

We earnestly appreciate Editors/Reviewers’ hard work and hope that the corrections will meet with approval.

Once again, thank you very much for your comments and suggestions.
