# Supplementary material for: Transcriptome analysis of Actinidia chinensis in response to Botryosphaeria dothidea infection
Source: PLoS One. 2020 Jan 8;15(1):e0227303. doi: 10.1371/journal.pone.0227303 (PMC6948751; doi:10.1371/journal.pone.0227303)
Supplement: Attachment — Submitted filename: Response to reviewers 2.docx [file pone.0227303.s015.docx]

**Comments from Reviewer** **2**

Q: I recommend the authors to use Storey's FDR method for controlling FDR because the BH method is too conservative; i.e., more false negatives. Storey's FDR method has been commonly accepted as a better alternative to the BH method.

A：Thank you very much for your comments. The Storey method you mentioned is indeed better than Benjamin and Hochberg method. In this manuscript, we choose Benjamin and Hochberg method because of these following reasons.

1. The screening of differential genes was completed by Beijing Novel Bioinformatics Co., Ltd. (https://en.novogene.com/), and the software used by this company was DESeq, which is still being used in this company. The calculation method used in the software is Benjamin-Hochberg method instead of Storey method. Actually, many transcriptome articles used this software and Benjamin-Hochberg method to do the calculation. Please see the following screenshots and references for detail. From the published papers, we can see that Benjamin-Hochberg method was adopted as long as DESeq or DESeq2 software was used to do the calculation. Obviously, these published papers demonstrated that Benjamin-Hochberg method is reliable and accepted by most journals.

**Fig. 1**

Ji, Q., Xu, X., Kang, L., Xu, Y., Xiao, J., & Goodman, SB., et al. 2019. Hematopoietic PBX-interacting protein mediates cartilage degeneration during the pathogenesis of osteoarthritis. Nat Commun. Jan 18;10(1):313. (<https://doi.org/10.1038/s41467-018-08277-5>.)

**Fig. 2**

Kang, W., Li, X., Sun, A., Yu, F., Hu, X. 2019. Study of the Persistence of the Phytotoxicity Induced by Graphene Oxide Quantum Dots and of the Specific Molecular Mechanisms by Integrating Omics and Regular Analyses. Environ Sci Technol. 2019 Apr 2;53(7):3791-3801. ([https://doi.org/10.1021/acs.est.8b06023. Epub 2019 Mar 21](https://doi.org/10.1038/s41467-018-08277-5).)

**Fig. 3**

Hu, P., Wang, T., Liu, H., Xu, J., Wang, L., & Zhao, P., et al. (2019). Full-length transcriptome and microRNA sequencing reveal the specific gene-regulation network of velvet antler in sika deer with extremely different velvet antler weight. Molecular Genetics and Genomics. 294(2),431-443. (<https://doi.org/10.1007/s00438-018-1520-8>)

2. The focus of this manuscript is in the experimental part and results and discussion, not in the comparison of different methods. Therefore, the algorithm used by most transcriptome articles was chose when we analyzed the data.

3. Furthermore, we also measured the fluorescence quantitative values of three differentially expressed genes (Fig.4). We found that the trends of the fluorescence quantitative values data are consistent with the data obtained by differential genes calculation, which indicated that the results obtained by this Benjamin-Hochberg method were reliable.

**Fig. 4**
